# Impact of the Grain for Green Project on water resources and ecological water stress in the Yanhe River Basin

**DOI:** 10.1371/journal.pone.0259611

**Published:** 2022-06-16

**Authors:** Yuping Han, Fan Xia, Huiping Huang, Wenbin Mu, Dongdong Jia

**Affiliations:** 1 College of Water Resources, North China University of Water Resources and Electric Power, Zhengzhou, Henan, China; 2 Henan Key Laboratory of Water Resources Conservation and Intensive Utilization in the Yellow River Basin, Zhengzhou, China; 3 School of Water Conservancy, North China University of Water Resources and Electric Power, Zhengzhou, China; Universiti Sains Malaysia, MALAYSIA

## Abstract

The Grain for Green project (GGP), initialized by the Chinese government in 1999, has achieved substantial achievements accompanied by a decrease in surface runoff on the Loess Plateau, but the impacts of large-scale afforestation on regional water resources are uncertain. Hence, the objective of this study was to explore the impact of land use change on generalized water resources and ecological water stress using the blue and green water concepts, taking the Yanhe River Basin as the case study. The Soil and Water Assessment Tool (SWAT) was applied to quantify the green water and blue water, which are defined as generalized water resources. The ecological water requirement of vegetation (forest and grass), agricultural water footprint and virtual water flow are considered regional water requirements. The land use types of 1980 (Scenario I) and 2017 (Scenario II) were entered into the SWAT model while keeping the other parameters constant to isolate the influence of land use changes. The results show that the average annual differences in blue, green and generalized water resources were -72.08 million m^3^, 24.34 million m^3^, and -47.74 million m^3^, respectively, when the simulation results of Scenario II were subtracted from those of Scenario I, which shows that land use change caused by the GGP led to a decrease in blue and generalized water resources and an increase in green water resources. Surface runoff in Scenario I was more than that in Scenario II in all of the years of the study period from 1980–2017, and green water storage in Scenario I was more than that in Scenario II in all of the years of the study period except in 1998; although lateral flow in Scenario I was less than that in Scenario II except in 2000 and 2015, as was groundwater runoff in 1992, 2000 and 2015, and green water flow in 1998. Blue water flow, green water storage and green water flow in Scenario II were less than those in Scenario I in the whole basin, 12.89 percent of the basin and 99.21 percent of the basin, respectively. The total water footprint increased from 1995 to 2010 because the forest water footprint increased significantly in this period, although the agricultural water footprint and grass water footprint decreased. The ecological water stress index values had no obvious temporal change trends in either land use scenario, but the ecological water stress index in Scenario II was greater than that in Scenario I, which illustrates that the GGP led to an increase in ecological water stress from the perspective of generalized water resources.

## Introduction

The Chinese government launched the Grain for Green project (GGP) in the 1990s to control soil erosion and water loss in the Loess Plateau [1–3]. After more than two decades of vegetation restoration, soil erosion caused by unreasonable land use has been curbed, and the ecological environment of the region have been greatly improved [4, 5]. Theoretically, vegetation restoration can enhance vegetation coverage, increase precipitation interception and water retention, decrease soil erosion and thus improve ecosystem stability. However, forests consume more water than other vegetation types, such as agricultural crops and natural grasslands [6]; therefore, accompanied by the enhancement of vegetation coverage since 2000 in China, runoff has shown a significant decrease in Haihe, Liaohe, Songhua Jiang, Hanjiang and the Yellow River [7–9]. In particular, runoff in the middle reaches of the Yellow River, which has the most obvious vegetation restoration achievements, has decreased sharply, and the annual flow at the Huayuankou station decreased from 55.9 billion m^3^ (1970s) to 45.2 billion m^3^ (2010–2015) [10]. Some results indicate that large-scale vegetation restoration on the Loess Plateau has positive impacts on soil erosion control and the ecological environment and negative impacts on streamflow [11–13]. Studies in other areas of the world have also demonstrated that an increase in vegetation coverage will lead to higher interception loss, which is the main reason for streamflow reductions [14, 15]. Some studies have also indicated that unsuitable vegetation types and overlooking biodiversity will bring about soil desiccation on the Loess Plateau [16, 17]. Studies have also presented the negative effects of afforestation on underground water resources [18] and ecological water deficits because of afforestation [19]. Why do streamflow decrease and where does the water go? Has the soil become dryer and has water stress become more serious because of the GGP? These are important problems need to be discussed.

The blue and green water concepts proposed by Falkenmark [20] provide new theories and ideas for water resource management, especially in arid and semiarid regions. A large amount of blue water converting to green water is one of the important causes of the streamflow decline on the Loess Plateau [21]. Vegetation coverage improvement significantly reduces streamflow, and vegetation restoration is close to the threshold of the water resources carrying capacity from the perspective of blue water [22]. However, it can reduce the water in sediment transport, and the virtual water embodied in green plants is far greater than streamflow reduction from the perspective of green water [23]. The water footprint (WF) proposed by Hoekstra and Hung [24] represents direct and indirect measurements of water appropriation by human beings. It quantifies blue and green water consumption in a river basin or a specified region and is a new approach to assess the sustainable water use of economic production sectors or regions [25–27].

The Yanhe River Basin in the Loess Plateau is the first tributary of the Yellow River and is in a semi-arid area having serious water scarcity and severe soil erosion. This watershed was one of the earliest and fastest areas in the whole country to convert cultivated land (grasslands) to forests and the vegetation restoration effect has been significant since the implementation of the GGP. During the period of 2000–2017, forests increased by 2357.6 km^2^, cultivated land decreased by 2116.2 km^2^, urban land increased by 222.1 km^2^, and water, grassland and other land cover types did not change remarkably. Numerous studies have attempted to evaluate the impact of vegetation restoration on water resources and have focused on improving the watershed ecological environment, ameliorating soil properties, and changing streamflow and sediment [28–30]. However, there has been almost no study exploring the impact of vegetation restoration on water stress from the aspect of the water footprint. The WF can assess natural water resource availability, support optimal allocation among different regions and improve watershed sustainability. It can evaluate water demand and consumption more comprehensively from the perspective of blue water, green water and WF. Therefore, the objectives of this study were to (1) analyze the spatial-temporal characteristics of land use change during the period of 1980–2017 in the Yanhe River Basin and describe GGP achievements; (2) quantify water balance elements and analyze the spatial-temporal characteristics of green and blue water over the whole basin and subbasins based on calibrated and validated SWAT model simulation results; (3) investigate the temporal characteristics of agricultural WF, ecological WF and virtual water flow; and (4) calculate the ecological water stress index based on generalized water resources and the water footprint and probe the impact of GGP on regional water stress.

## Materials and methods

### Study area

**Fig 1:**
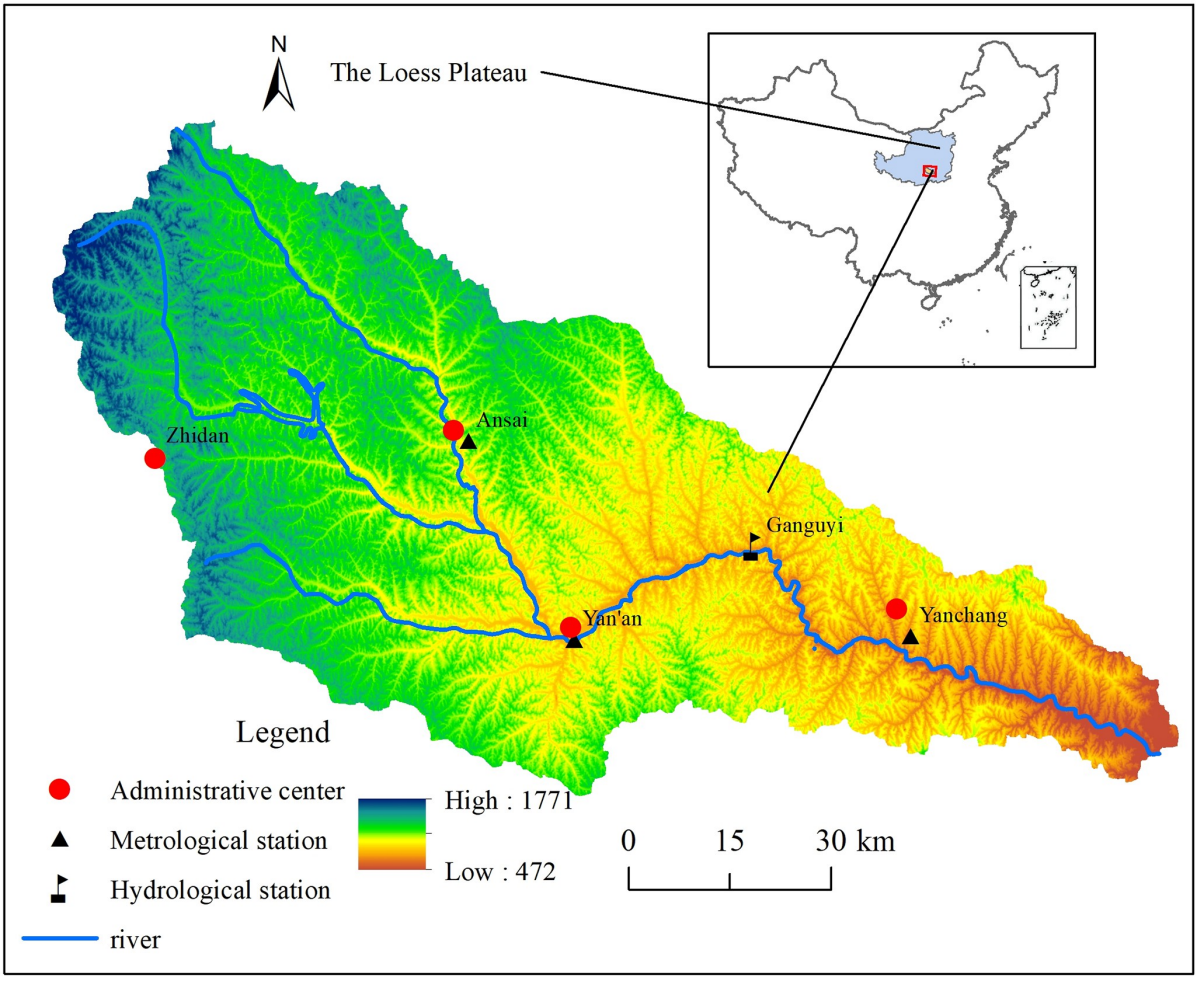
Location and distribution of the hydrological and meteorological stations in the Yanhe River Basin. Yanhe river watershed (right; red square) in China, meteorological station locations marked by black triangles. The blue line indicate yanhe river. Red dots indicate administrative center and black flag indicate hydrologic station. The Yanhe river catchment (left) and elevation range. Digital elevation model data source: the Geospatial Data Cloud (http://www.gscloud.cn/). The map was prepared in ARCGIS using basin boundaries from the China Database of Topographic map.

The Yanhe River Basin (36.21’-37.19’N, 108.38’-110.29’E) is located on the Loess Plateau in northern Shaanxi Province in China and is a first-order tributary of the Yellow River (Fig 1). With an area of 7785 km^2^, the watershed has a warm temperate continental semiarid monsoon climate. The annual mean precipitation of the watershed is 520 mm, with 75% being concentrated from June to September, and the mean annual temperature varies from 8.8 to 10.2 degrees Celsius [31]. The yearly streamflow at Gan’guyi was 220 million m^3^, and the major land use and land cover types of the watershed were forestland, shrub land, grassland, cultivated land, construction land, water body, and bare land. The annual water resource per capita value in the basin was 375 m^3^, accounting for 28 percent of that of Shaanxi Province and 17 percent of that of China. This river has a large sediment content and serious point source and nonpoint source pollution. The water resources in the watershed are in acute shortage, and the ecological environment is fragile.

### Data sources

The land uses of the region in 1980 (before the GGP) were obtained from the Resource and Environment Science and Data Center (http://www.resdc.cn), and the land uses of 2017 (after the GGP) were interpreted from Landsat OLI images. The image data having 30 m resolution and zero cloud cover were collected from the Geospatial Data Cloud (http://www.gscloud.cn/). The land uses of 1990, 2000, 2005, and 2010 were also from the Resource and Environment Science and Data Center (http://www.resdc.cn). The land use types in the Yanhe River basin are classified into cultivated land, forest, grass, urban use land, waters and barren land. Digital evaluation model (DEM) data with 30 m resolution were provided by the Geospatial Data Cloud (http://www.gscloud.cn/). The daily meteorological data were extracted from China’s meteorological data sharing service system (http://cdc.cma.gov.cn/home.do). Soil data with an accuracy of 1:1000000 were obtained from the Data Center for Resources and Environmental Sciences (http://vdb3.soil.csdb.cn/). Annual and monthly streamflow data of the hydrological stations were provided by the Loess Plateau SubCenter, National Earth System Science Data Center, National Science & Technology Infrastructure of China (http://loess.geodata.cn). Various crop yields, populations, and grain consumption were obtained from Yan’an Statistical Yearbooks (1995–2018), which were published by China Statistics Press; forest and grassland areas were extracted from land use maps.

### SWAT model

SWAT is a physically based, semidistributed model that has the advantage of simulating the quality and quantity of both surface and ground water as well as predicting the impact of land cover change, land management practices and climate change [32–34]. The model has been successfully applied to calculate water yield and evaluate water quality in small watersheds and large river basins [35, 36]. Simulation results can be applied to quantify the spatial and temporal characteristics of blue and green water in different parts of the world, such as the Savannah River Basin in the southeast Atlantic region of the USA [37], the Weihe River in northwest China [38], and the Athabasca River Basin in Canada [39].

The sequential uncertainty fitting (SUFI-2) algorithm (Abbaspour, 2015) [40, 41] in SWAT calibration and uncertainty programs (SWAT-CUP) were used to perform parameter estimation and sensitivity analysis. The algorithm describes all parameters and tries to simulate the observed data within the 95% confidence interval (95 PPU) by using an iterative process. The primary target of calibration is to identify the sensitive parameters in the watershed that control surface runoff. The P-factor and R-factor are used to quantify the uncertainty in this algorithm. The P-factor measures the percentage of the measured data with ninety-five percent prediction certainty. The R-factor represents the average distance of 95 PPU divided by the standard deviation of the measured data. P-factor values range between 0 and 1, and the R-factor values range from 0 to infinity; the closer the P-factor is to 1 and the closer the R-factor is to 0, the better the calibration and uncertainty analysis results [42]. R^2^ (the coefficient of determination) and NSE (the Nash-Sutcliffe efficiency) are used to evaluate the accuracy of the SWAT model. R^2^ values range from 0 to 1, and if the value is close to 1, it indicates that the simulated data are close to the observed data and vice versa [43].

### Estimating blue and green water resources

Blue water resources in each subbasin are equal to water yield (WYLD), which is the sum of surface runoff (SURQ), lateral flows (LATQ) and ground water recharge (GWQ) [44]. Green water resources consist of green water flow (actual evapotranspiration, ET) and green water storage (soil water content, SW). The annual SURQ, LATQ, GWQ, ET and SW were obtained from simulation results using the calibrated and validated SWAT model [45, 46].

To investigate the influences of land use change caused by the GGP on blue, green water and each hydrological element, two scenarios are set up by changing land use types while keeping the other input parameters in SWAT model unchanged (scenario I: land uses of 1980; scenario II: land uses of 2017). Only the impact of land use changes was considered when the simulation results of scenario II were subtracted from those of scenario I.

### WF

The WF concept quantifying the volumetric freshwater consumption of products is distinguished as green, blue and gray WFs [20]. Green WF (WFgreen) is defined as rainwater that is stored in the soil and evaporated or consumed during production. Blue WF (WFblue) refers to surface and groundwater that are consumed or evaporated during production. The gray water footprint (WFgray) expresses the volume of water needed to dilute pollutants to achieve allowed values in the receiving water bodies. The sum of WFgreen and WFblue demonstrates the quantity of total freshwater consumption during production, while WFgray indicates the degradation of water quality. The goal of this study has been to explore water quantity changes caused by vegetation restoration, so WFgray is not considered during the calculation process.

#### Agricultural crop WF

The crop WF is the sum of WF_green_ and WF_blue_, which are determined by the following equations [47]:

$$WF_{green}=\frac{CWU_{green}}{Y}=10\times \frac{EY_{green}}{Y}$$ (1)

$$WF_{blue}=\frac{CWU_{blue}}{Y}=10\times \frac{ET_{blue}}{Y}$$ (2)

where *WF*_*green*_ and *WF*_*blu*e_ are the green WF (m^3^/t) and blue WF (m^3^/t) during the crop growth season, respectively; *CWU*_*green*_ and *CWU*_*blue*_ are the green and blue water uses (m^3^/ha); 10 is a constant to convert the water depth (mm) to the water volume (m^3^/ha); *Y* is the crop yield (m^3^/ha); and *ET*_*green*_ and *ET*_*blue*_ are defined as the evaporative demand satisfied by green and blue water, respectively. *ET*_*green*_ and *ET*_*blue*_ are calculated as [48]:

$$ET_{green}=\text{min}(\begin{array}{ccc} ET_{c} & , & P_{eff} \end{array})$$ (3)

$$ET_{blue}=\text{max}(\begin{array}{ccc} 0 & , & ET_{c}-ET_{green} \end{array})$$ (4)

where *ET*_*c*_ represents the actual evapotranspiration of crops from sowing day to the harvest and *P*_*eff*_ is the effective precipitation [49].

$$ET_{c}=K_{c}\times ET_{0}$$ (5)

$$P_{eff}=\{\begin{array}{ll} P(4.17-0.02P)/4.17 & P<83\text{mm}\\ 41.7+0.1P & P\geq 83\text{mm} \end{array}$$ (6)

where *K*_*c*_ is the crop coefficient, *ET*_*0*_ is the potential reference crop evapotranspiration and is calculated by the Penman–Monteith formula, and *P* is the precipitation of ten days.

#### Agricultural virtual water flow calculation

Due to the lack of statistics on regional trade and storage of agricultural products in China, the calculation of agricultural virtual water flow in this study was based on the assumption that the regional agricultural product storage remains unchanged and the water footprints per unit mass of local agricultural product inputs and outputs are equal. The equation is as follows [50]:

$$W_{v,f}=w_{v,\text{pro}}-W_{v,\text{con}}$$ (7)

where *W*_*v*,*f*_ is the regional virtual flow; while *W*_*v*,*pro*_, and *W*_*v*,*con*_ are the amounts of virtual water production and water consumption, respectively. *W*_*v*,*pro*_ and *W*_*v*,*con*_ can be calculated by multiplying the agricultural virtual water per unit mass times the amounts of grain production and grain consumption, respectively. If *W*_*v*,*f*_ is less than 0, it indicates that the region inputs virtual water from outside; if *W*_*v*,*f*_ is greater than 0, it indicates that the region exports virtual water, which may aggravate regional water resources pressure; if is *W*_*v*,*f*_ is equal to 0, it indicates that there is no transfer of agricultural products between the region and the outside.

#### Vegetation WF

The equation of vegetation WF is [51]:

$$W_{Fvegetation}=\sum_{p=1}^{n}A_{p}\times ET_{p}$$ (8)

where *WF*_*green*_ is the vegetation WF (m^3^); *A*_*p*_ is the area of vegetation coverage (m^2^); *ET*_*p*_ is the vegetation evapotranspiration (mm/day) under restricted circumstances; and *p* is the vegetation type.

Vegetation evapotranspiration is less than the potential evapotranspiration when the soil water content is below a specified threshold, and the effect is determined by the soil moisture limitation coefficient (*K*_*s*_). Thus, the calculation equation of *ET*_*p*_ is as follows [52]:

$$ET_{p}=\sum_{j=1}^{n}ET_{0}\times K_{c}\times K_{sj}$$ (9)

where *ET*_*0*_ is the potential reference evapotranspiration, *K*_*c*_ is the vegetation water demand coefficient, *K*_*s*_ is the soil moisture limitation coefficient, *and j* is the soil type.

Based on previous studies on the Loess Plateau [53, 54], the *K*_*c*_ values of forest and grassland are 0.765 and 0.65, respectively. The Yanhe River Basin contains silty soil and sandy loam soil, and the *K*_*s*_ values of the two soil types are 0.537 and 0.556, respectively.

### Ecological water stress index (EWSI)

**Fig 2:**
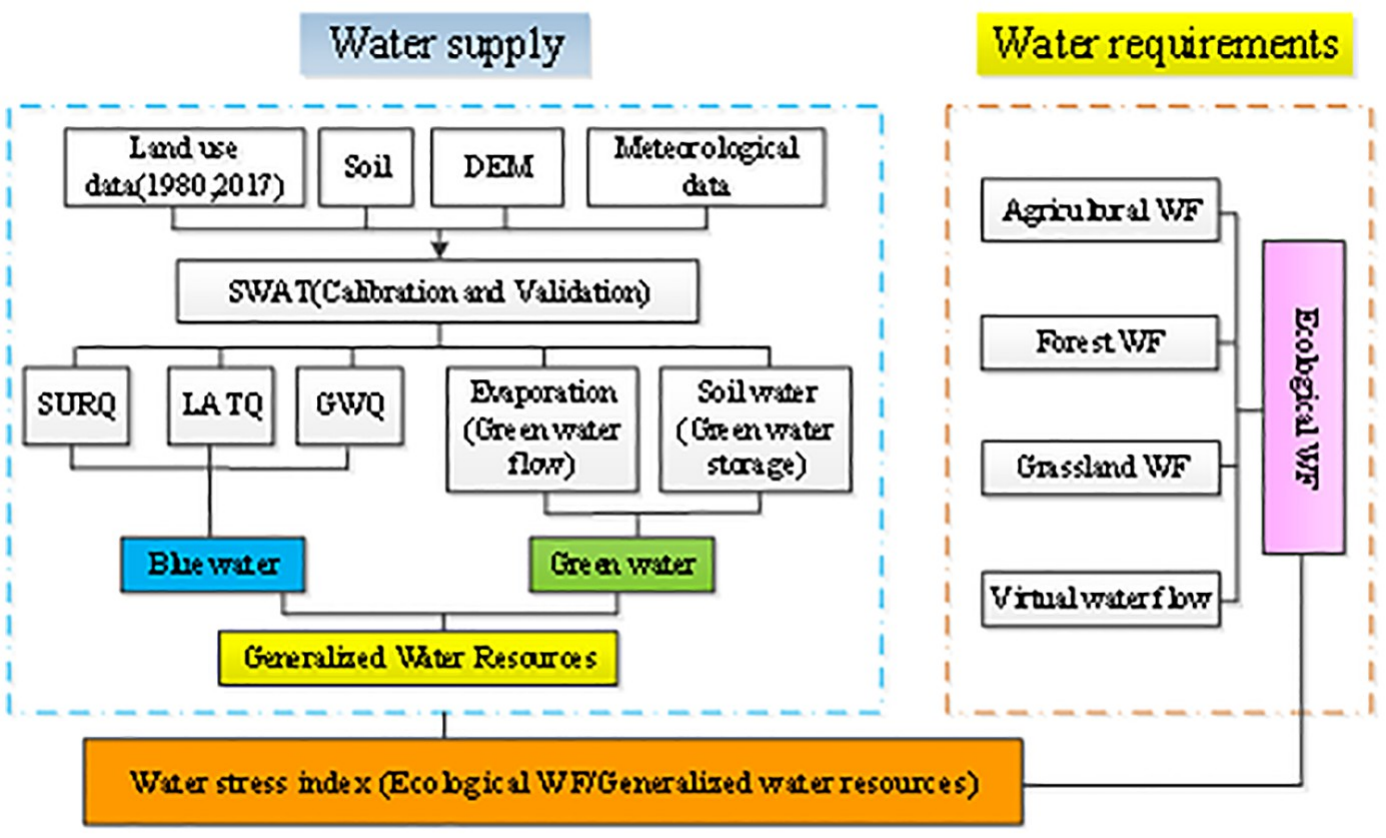
Framework for assessing the ecological water stress in the Yanhe River Basin.

Raskin [55] proposed a criterion using the ratio between water demand and available water resources to estimate water scarcity, and it has been widely used to evaluate global and regional water resources [56, 57]. The water stress index (WSI) can provide information on the management of freshwater resources [58]. In this paper, the EWSI is calculated as the ratio of the ecological water footprint to generalized water resources (Fig 2). Generalized water resources are the sum of blue water and green water simulated by the SWAT model, and the ecological water footprint, which contains the agricultural WF, forest WF, grass WF and virtual water flow.

## Results

### Land use change in Yanhe River Basin

**Fig 3:**
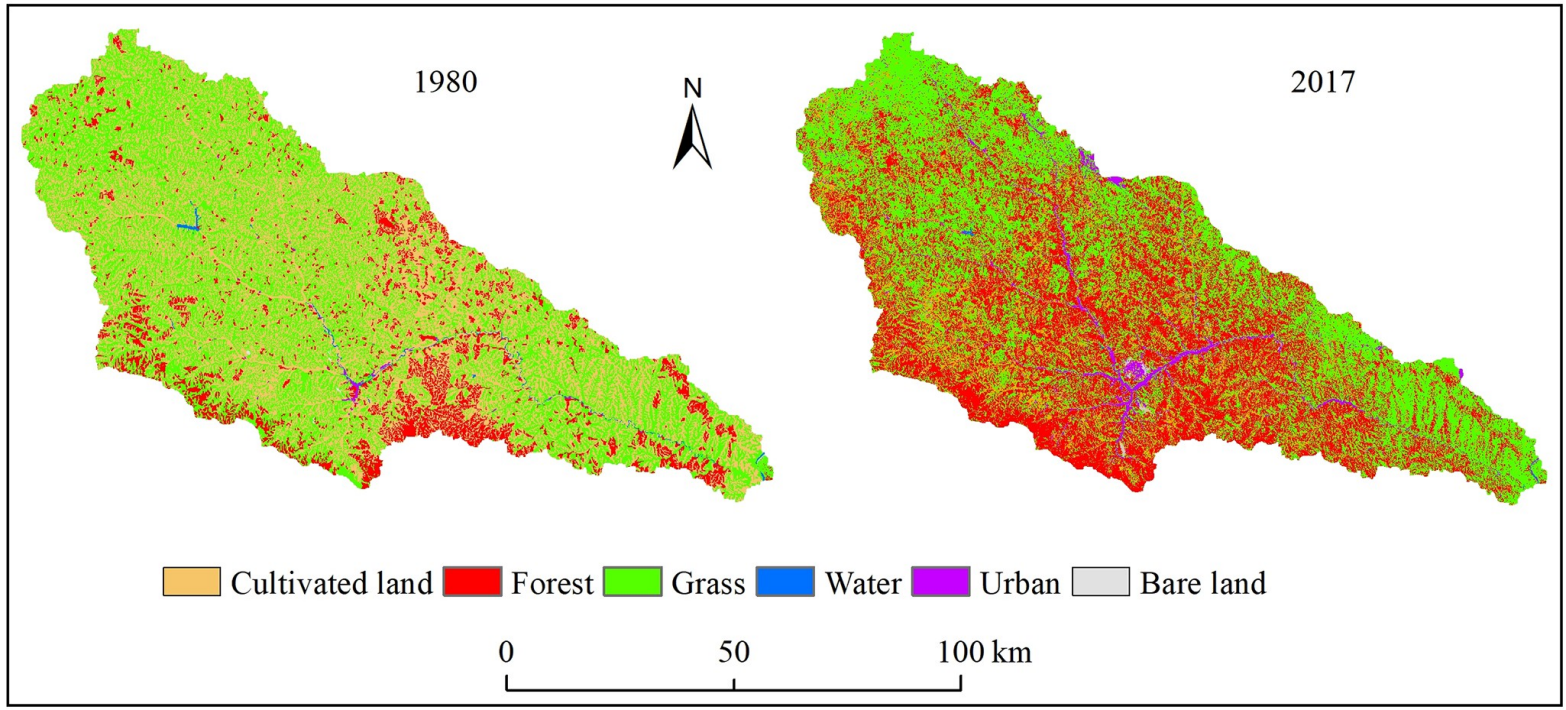
Land use map of the Yanhe River Basin in the different years. The map was prepared in ARCGIS using Classification of land use types from the Resource and Environment Science and Data Center (http://www.resdc.cn). The land uses of 2017 were interpreted from Landsat OLI images which were collected from the Geospatial Data Cloud (http://www.gscloud.cn/).

The Yanhe River Basin land use types of 1980 and 2017 are shown in Table 1 and Fig 3. There are no large areas of cultivated land, and the use types are scattered between grasslands and woodlands. Grassland was located in the upper and lower reaches of the basin in both 1980 and 2017, and forestland was located in the middle reaches in 2017. The dominant land use types were grass and cultivated land, which accounted for approximately 88.82% of the whole area in 1980, while grassland and forestland accounted for approximately 84.33% of the whole area in 2017. There were two obvious land use changes from 1980 to 2017: increases in forestland and urban use land and decreases in cultivated land, grassland and water. Compared with the land use in 1980, cultivated land area changed from 3348.9 km^2^ to 872.10 km^2^, and the area percent decreased from 43.02 to 12.20. Forestland area changed from 782.4 km^2^ to 3018.52 km^2^, and the area percent increased from 10.05 to 38.77. The area percentages of grassland, water, barren land, and urban use land changed slightly, and their area percent values changed by -0.24, -0.20, 0.32 and 3.2, respectively. Land use change demonstrates that the GGP obtained great achievements in the Yanhe River Basin.

**Table 1:** Land uses in the Yanhe River Basin in 1980 and 2017.

Year	Land use	Cultivated land	Forest	Grass	Urban	Waters	Barren
**1980**	Area(km^2^)	3348.90	782.40	3565.20	12.60	26.90	49.00
	Percent(%)	43.02	10.05	45.80	0.16	0.35	0.63
**2017**	Area(km^2^)	872.10	3018.52	3547.09	261.84	11.42	74.03
	Percent(%)	11.20	38.77	45.56	3.36	0.15	0.95
**change**	Area(km^2^)	-2476.80	2236.12	-18.11	249.24	-15.48	25.03
	Percent(%)	-31.82	28.72	-0.24	3.20	-0.20	0.32

The diagonal value in the land use conversion matrix (Table 2) is the unchanged area of each land use type. 5132.30 km^2^ of land use type has changed during the period from 1980 to 2017, 2937.70 km^2^ of cultivated land has changed into other land use types and the area proportion is 57.24%; 1198.30 km^2^ has been converted into forest and 1586.44 km^2^ has been converted into grassland. Grassland is the second land use conversion type (1843.56 km^2^) and it was mainly converted into forest (1337.85 km^2^), cultivated land (388.01 km^2^) and urban use land (103.85 km^2^). The transfer-out land use type was cultivated land and grassland, and the transfer proportion accounted for as high as 93.16% of the whole basin.

**Table 2:** Transition matrix of land use conversions (km^2^) from 1980 to 2017.

**2017**									
	**Land use type**	**Cultivated land**	**Forest**	**Grass**	**Urban**	**Waters**	**Barren**	**1980**	
								**Sum**	**%**
**1980**	**Cultivated land**	453.14	1198.30	1586.44	138.84	1.04	13.08	2937.70	57.24
	**Forest**	65.98	479.30	226.26	13.40	0.23	3.08	308.94	6.02
	**Grass**	388.01	1337.85	1710.36	103.85	2.67	11.19	1843.56	35.92
	**Urban**	3.41	5.00	8.04	9.77	1.82	0.11	18.38	0.36
	**Waters**	3.07	3.10	3.49	11.51	0.12	0.05	21.23	0.41
	**Barren**	0.43	0.81	1.22	0.03	0.00	0.00	2.49	0.05
**2017**	**Sum**	460.90	2545.06	1825.45	267.62	5.75	27.52	5132.30	
	**%**	8.98	49.59	35.57	5.21	0.11	0.54		

Note: the sums of the rows and columns are exclusive of the unchanged areas of each land use type

A total of 2545.06 km^2^ of forest was developed from other land use types since 1980, and most of the conversion was from grass land and cultivated land; in the area transferred from other land use types, the proportion of forest accounts for 49.59%. A total of 1825.45 km^2^ of grassland was from other land use types, and 1198.30 km^2^ was from cultivated land; the grassland transfer proportion is 35.57% of the whole basin. Although cultivated land was mostly transferred into forestland and grassland, 65.98 km^2^ and 388.01 km^2^ of cultivated land was from forestland and grassland, respectively.

### Calibration and validation of the SWAT model

The sensitivity analysis indicates that CN2 (SCS streamflow curve number for moisture Condition 2), CANMX (Maximum canopy storage), ALPHA_BNK (Baseflow alpha factor for bank storage), SOL_AWC (Available water capacity of the soil layer), SOL_K (Saturated hydraulic conductivity of the soil layer), SOL_BD (Moist bulk density of the soil layer), ESCO (Soil evaporation compensation factor), and REVAPMN (Threshold depth of water in the shallow aquifer for "revap" to occur) are more sensitive than the other parameters of the streamflow simulation. The sensitive parameters of streamflow in this paper are in accord with the summary for streamflow in the SWAT simulation of Athira (2021) [59], and their optimal values for the SWAT model are shown in Table 3.

**Table 3:** The initial ranges and final values of the sensitivity parameters.

Parameters	Rank	t-Stat	Calibrated method	P Value	Initial range	Optimal value
CN2.mgt	1	-33.69	R	0.00	(-1,1)	-0.74
CANMX.hru	2	6.02	V	0.00	(0,100)	0.04
ALPHA_BNK. rte	3	-4.28	V	0.00	(0,1)	0.11
SOL_AWC.sol	4	3.81	R	0.00	(-1,1)	-0.76
SOL_K.sol	5	-3.69	R	0.00	(-1,1)	0.13
SOL_BD.sol	6	-2.95	R	0.00	(-1,1)	0.16
ESCO.hru	7	-2.35	V	0.02	(0,1)	0.37
REVAPMN.gw	8	1.67	V	0.09	(0,500)	127.83

Note: R indicates that the existing parameter value was multiplied by (1+ a given value), V indicates that the default parameter was replaced by a given value.

**Fig 4:**
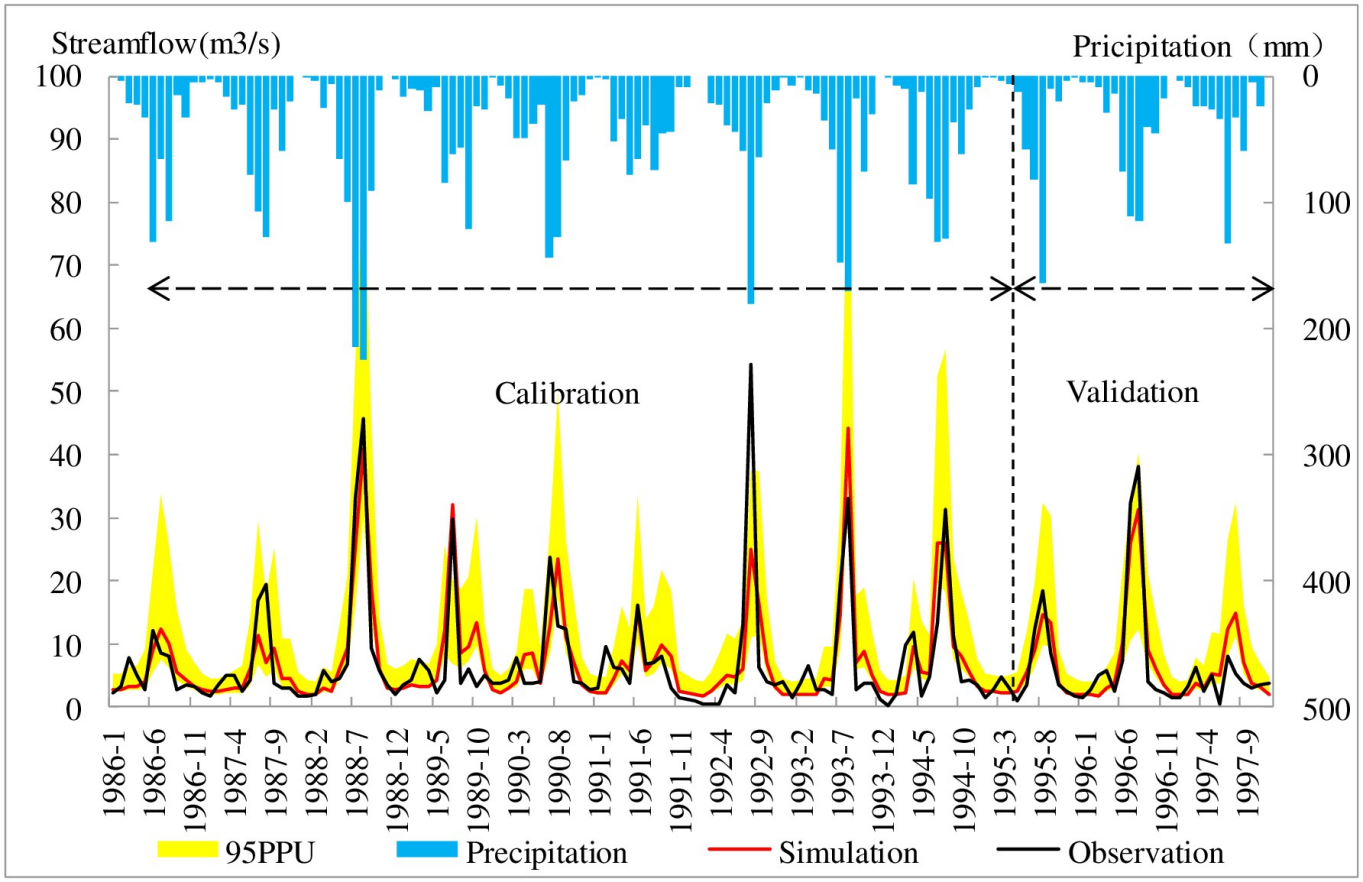
Time series plot between the observed and simulated streamflows at the monthly time scale and 95% prediction uncertainty bands.

SWAT-CUP is used to calibrate and validate the model in this study; 1980–1985 was selected as the warm-up period, 1986–1994 was the calibration period, and 1995–1997 was the validation period. R^2^ (the coefficient of determination), NSE (the Nash-Sutcliffe efficiency coefficients), BIAS (percent bias), P-factor and R-factor are used to assess the accuracy of the model simulation. When the R^2^ and NSE values are more than 0.5, the BIAS value is less than or equal to ±20%, the P-factor is more than 0.6 and the R-factor is less than 1.5, the SWAT calibration results on a monthly scale are considered acceptable [60]. Table 4 shows the calculation results of the model evaluation. Fig 4 illustrates the calibration and validation results at the Ganguyi hydrological station in the Yanhe River Basin. R^2^, NSE, BIAS, P-factor and R-factor are equal to 0.79, 0.73, 2.2%, 0.75 and 1.24 in calibration period; R^2^, NSE, BIAS, P-factor and R-factor are equal to 0.71, 0.69, 15%, 0.66 and 0.52 in validation period, respectively. The P-factors in the calibration and validation indicate that 75 and 66 percent of observed data are in the 95% confidence interval. The goodness-of-fit statistics indicate reasonable agreement between the observed and simulated streamflows. However, there was a higher deviation in July in 1989 and 1996 because the precipitation on the 16th of July in 1989 was 26.7 mm and the precipitation on the 12th of July in 1996 was 91.9 mm, which accounted for 49.7% and 98.9% of the July precipitation of those two years, respectively, which may be the reason for the comparatively higher deviations between the observed and simulated streamflows. The SWAT model cannot accurately simulate rainstorm processes, and the heavy rains of 1989 and 1996 led to the poor simulation of those two years and reduced the overall accuracy of the simulation and validation periods.

**Table 4:** Results of the SWAT model evaluation indicators in the Yanhe River Basin.

Periods	*R* ^*2*^	*NSE*	*BIAS*	*P-factor*	*R-factor*
Calibration	0.79	0.73	2.2%	0.75	1.24
Validation	0.71	0.69	15%	0.66	0.52

### Spatial-temporal change of blue and green water

#### Temporal change of blue and green water

- Interannual variations of blue and green water

**Fig 5:**
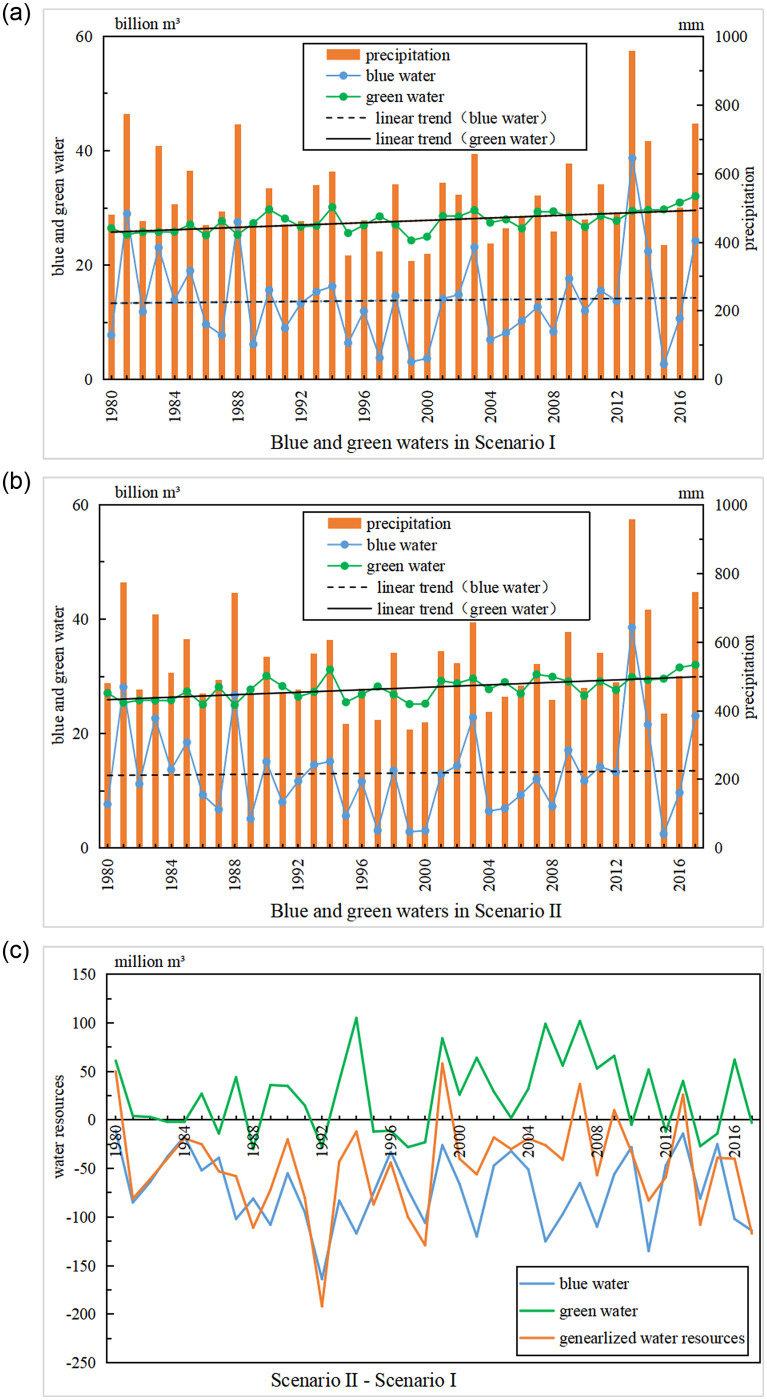
Time series changes of annual water resources.

The temporal change characteristics of blue and green waters under different vegetation cover conditions play an important role in the GGP policy. The annual blue and green waters simulated by the SWAT model in the two scenarios for the Yanhe River Basin are shown in Fig 5a and 5b. The annual average blue water resources are 13.71 billion m^3^, which accounts for 33.12% of the annual average total water resources, and the annual average green water resources are 27.68 billion m^3^, which account for 66.88% of the annual average total water resources in scenario I. The annual average blue water resources are 13.1 billion m^3^, and the annual average green water resources are 27.92 billion m^3^, which account for 31.93% and 68.07% of the annual average total water resources, respectively, in scenario II. The maximum blue water occurred in 2013 and the minimum in 1999, and the two years correspond to the maximum and minimum amounts of precipitation in the study period. Blue water values in 1981, 1988, and 2017 were greater than those of other years, and in 1995, 2000, and 2004, were less than those of other years, which is the same as the temporal characteristics of precipitation and indicating that the amounts of blue water were strongly related to rainfall, and the correlation coefficient of the two elements was 0.97. There is no significant relation between the amounts of rainfall and green water.

Blue water increases at rates of 0.87 million m^3^/yr and 2.2 million m^3^/yr in scenarios I and II, respectively; the green water increase rates are 10.29 million m^3^/yr and 10.8 million m^3^/yr in scenarios I and II, respectively. The increase rate of blue water in scenario II is approximately 2.52 times that of scenario I, but there is almost no difference in the increase rate of green water between the two different scenarios. Both blue water and green water show increasing trends in the two land use scenarios.

The blue water, green water and total water resources in scenario II were less than those in scenario I during years 38, 14 and 34 years of the 38 years studied (Fig 5c). The average annual differences in blue, green and total water resources between scenario II and scenario I are -72.08 million m^3^, 24.34 million m^3^, and -47.74 million m^3^, respectively, which indicates that land use change caused by the GGP leads to a decrease in blue and generalized water resources and an increase in green water resources.

- Interannual variations of the diverse hydrological components

**Fig 6:**
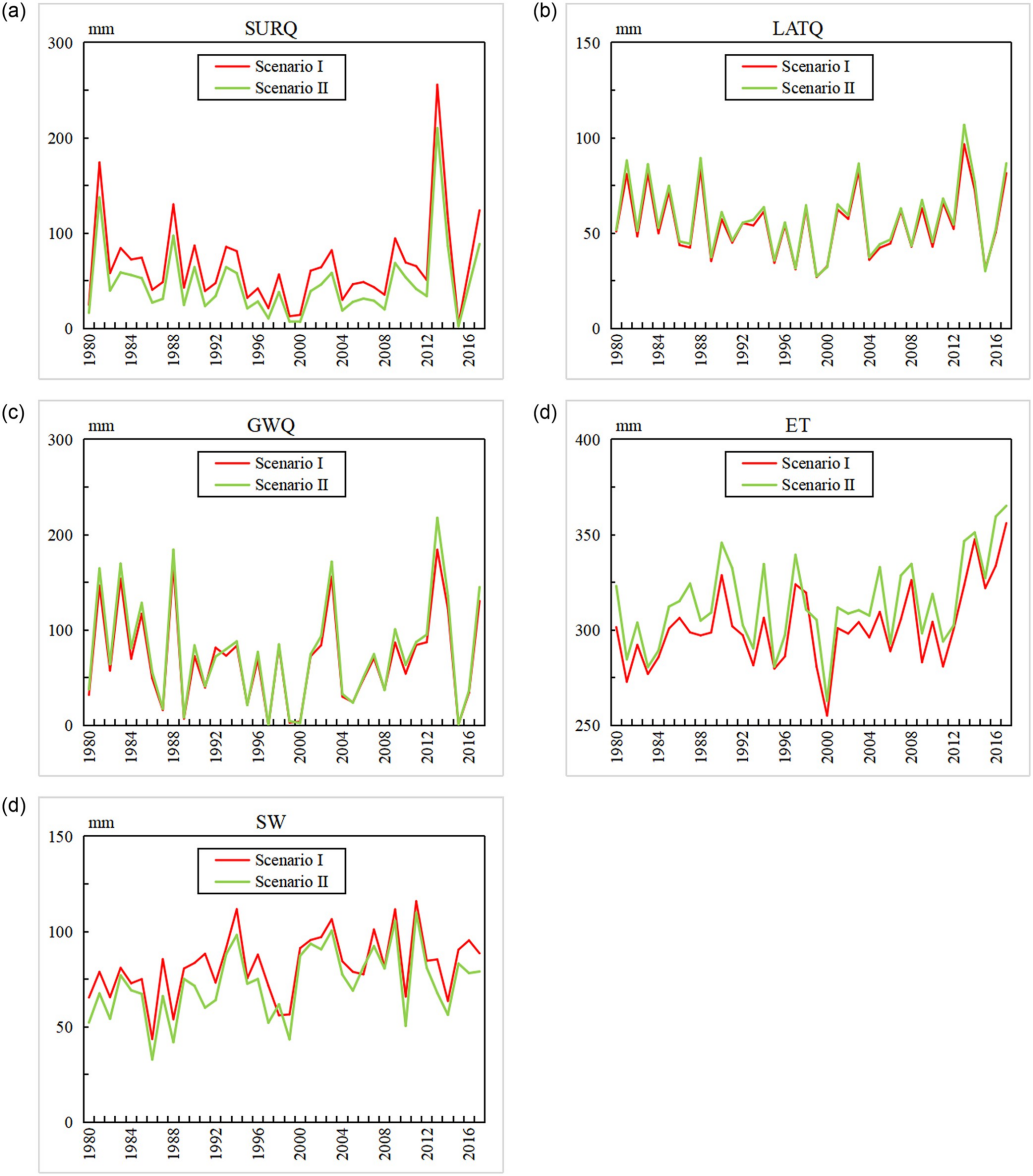
Temporal changes of the hydrological elements.

To clarify the impact of land use on regional water resources, it was necessary to analyze and quantify the diverse components of the hydrological elements within the study area. They include SURQ, LATQ, GWQ, ET, and SW obtained from the well-calibrated SWAT model. Fig 6 demonstrates that the annual SURQ and component of blue water in scenario II were smaller than those in scenario I in all of the studied years except 2017; the annual average difference between scenario II and scenario I is 17.56 mm, and the maximum difference of 45.61 mm appeared in 2013. The change in SURQ was consistent with the fact that surface runoff has decreased on the Loess Plateau since the implementation of the GGP. LATQ in scenario II was larger than that of scenario I in 2000 and 2015 but lesser in the other years; the change range of LATQ was the smallest of the five variables, the annual average difference between scenario II and scenario I was 2.5 mm. The GWQ in scenario II was larger than that in scenario I in 1992, 1995, 2000, 2005, 2008 and 2015 and smaller in the other years; the annual average difference between scenario II and scenario I was 6.57 mm.

The annual green water flow component of the green water in scenario II was larger than that of scenario I in all of the studied years except 2000, whereas the green water storage in scenario II was less than that of scenario I in all of the studied years (Fig 6). The mean annual green water flow had variations from 255.15 mm to 356.12 mm in scenario I and from 262.81 mm to 365.19 mm in scenario II; The mean annual green water storage had variations from 43.68 mm to 115.92 mm in scenario I and from 32.96 mm to 109.92 mm in scenario II throughout the study period; green water flows in scenario I were smaller than those of scenario II except in 1998, whereas green water storage values in scenario I were more than those of scenario II except in 1998. The differences of the green water flows between the two land use scenarios were comparatively larger than those of green water storage values. The annual average differences in green water flow and green water storage between scenario II and scenario I were 12.28 mm and -8.9 mm, respectively. The GGP reduced SURQ and green water storage but increases GWQ, LATQ and green water flow.

#### Spatial distribution changes of the blue and green water

**Fig 7:**
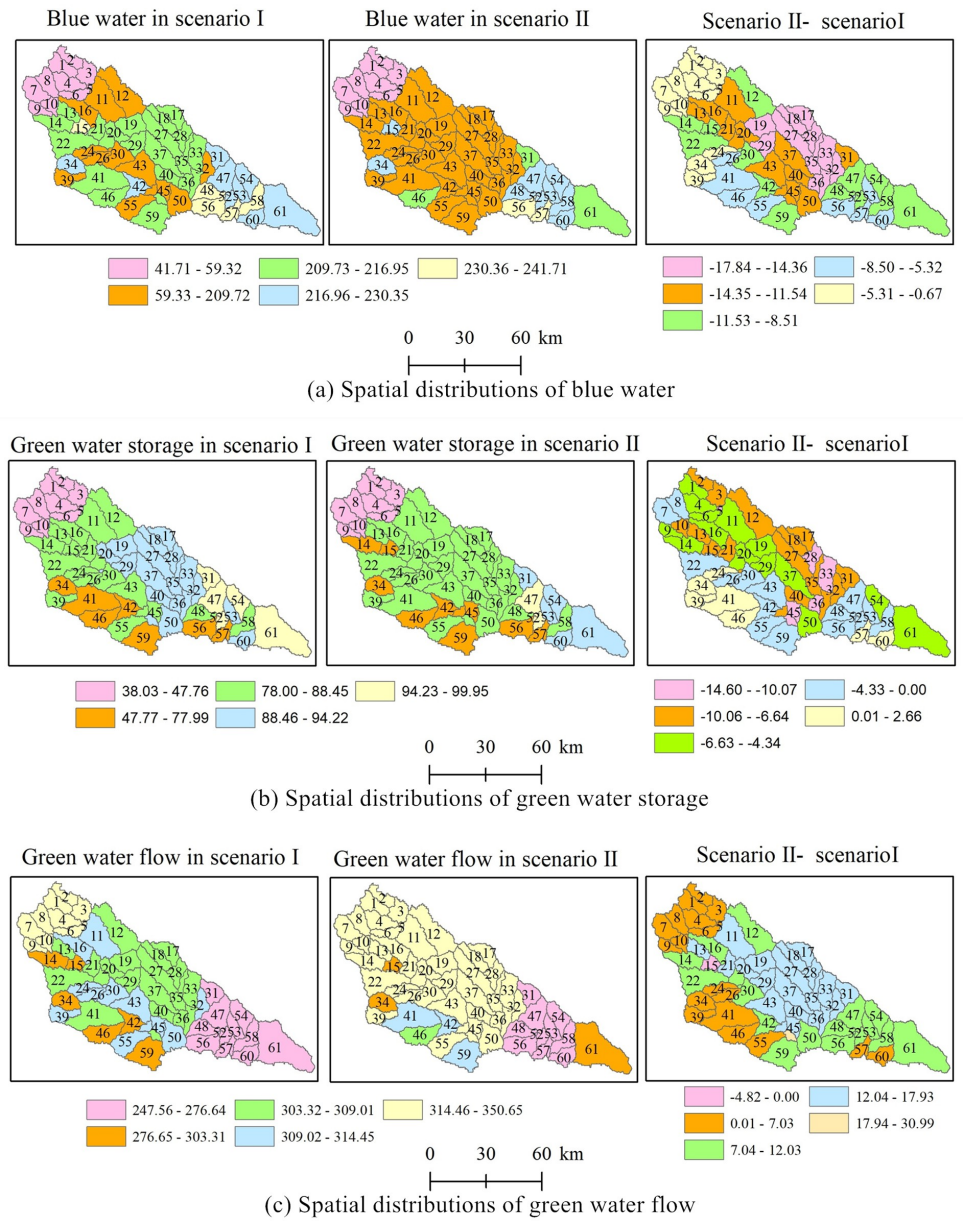
Spatial distributions of blue and green waters. The map was prepared in ARCGIS using sub-basin boundaries and blue/green water calculation results from the Results of watershed division on Yanhe river Basin. The spatial distribution range values of scenario 1 and scenario 2 were represented in the same color, and the spatial distribution range of their difference values was represented separately.

Blue water, green water storage, and green water flow had considerable spatial variations among subbasins in the two land use scenarios (Fig 7). Water resource values were divided into five ranks according to the natural breaks method in ArcGIS software in Scenario I, whereas the classification boundaries in Scenario II were the same as those used in Scenario I to compare the spatial variations of the two scenarios.

The spatial distributions of the mean annual blue water were less than 59.32 mm, which is exactly the same in the two scenarios, and the ten minimum change regions were distributed in the upper area of the basin (Fig 7a). It can be observed that blue water in the second rank jumps from 59.33 mm to 209.72 mm, 14 subbasins in Scenario I and 36 subbasins in Scenario II were in that range. Thirty-seven subbasins have average annual blue water values ranging from 207.93 mm to 241.71 mm, while only 15 subbasins were in that interval in Scenario II. Fig 7a shows that blue water in Scenario II was less than that in Scenario I in all subbasins, which demonstrates that blue water decreased over the whole basin with the implementation of the GGP. The minimum changes occurred in the upper part, and the maximum changes occurred in the middle part of the basin.

Fig 7b presents the green water storage across subbasins for the two scenarios. It indicates that the green water storage values lower than 47.76 mm were located in the upper reaches of the river basin in both of the two land use scenarios. The areas with values between 78 mm and 88.45 mm occupied 57.44 percent of the whole basin in Scenario II. Green water storage values in 8 subbasins in Scenario II were larger than those of Scenario I, and the area occupies 12.89 percent of the whole basin. The maximum decrease region is located in the northern and eastern parts of the basin, and the increase region is located in the western part of the basin.

The maximum green water flow values were located in the upper reaches in Scenario I but in the upper and middle reaches in Scenario II (Fig 7c). Data in 42 subbasins were more than 314.46 mm in Scenario II and the area accounted for 63.21 percent of the whole basin; green water flows lower than 276.64 mm accounted for 14.75 percent in Scenario II and 21.5 percent in Scenario I, indicating that there was comparatively little variability in areas of the minimum data range between two land use scenarios. Green water in one subbasin decreased, and its area was 0.79 percent of the basin; therefore, it can be regarded that the green water flow increased over the whole basin because of the GGP.

**Fig 8:**
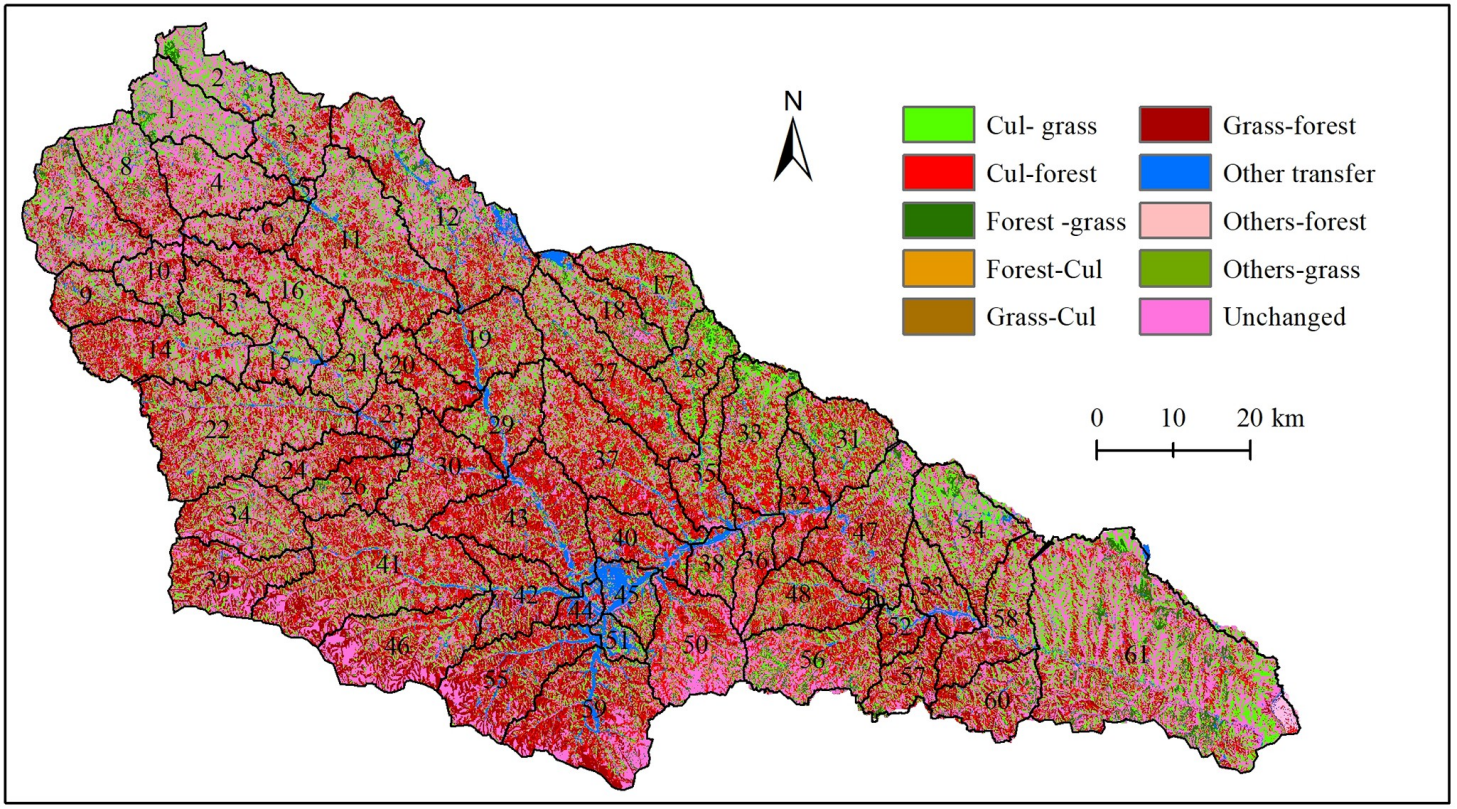
Land use conversions between 1980 and 2017. The map was prepared in ARCGIS using markov model.

The land use conversion map from 1980 to 2017 and subwatersheds in the SWAT model are superimposed on one map (Fig 8) to compare land use conversion with spatial changes in water resources. We can see that spatial changes in blue water, green water storage, and green water flow (Fig 7) are related to land use conversion to a certain extent. The areas with the lowest reduction of blue water were consistent with the areas in which land use remained unchanged, such as the subbasins from one to ten, whereas the areas with the largest reduction in blue water were consistent with the areas in which land use changed significantly, such as subbbasins 17, 18, 19, 27, 28, 29, 32, 33, 35, 36, 38, where large amounts of cultivated land were converted into grassland and forestland. The second reduction areas, such as subbasins 11, 13, 16, 20, 21, and 23, were also the areas in which many cultivated lands were converted into grassland and forestland. The relationship between the spatial changes in green water flows and land use conversions were relatively the same as those between blue water values and land use conversions. There was no significant relationship between land use conversions and spatial changes in green water storage.

### WF

#### Agricultural WF

**Fig 9:**
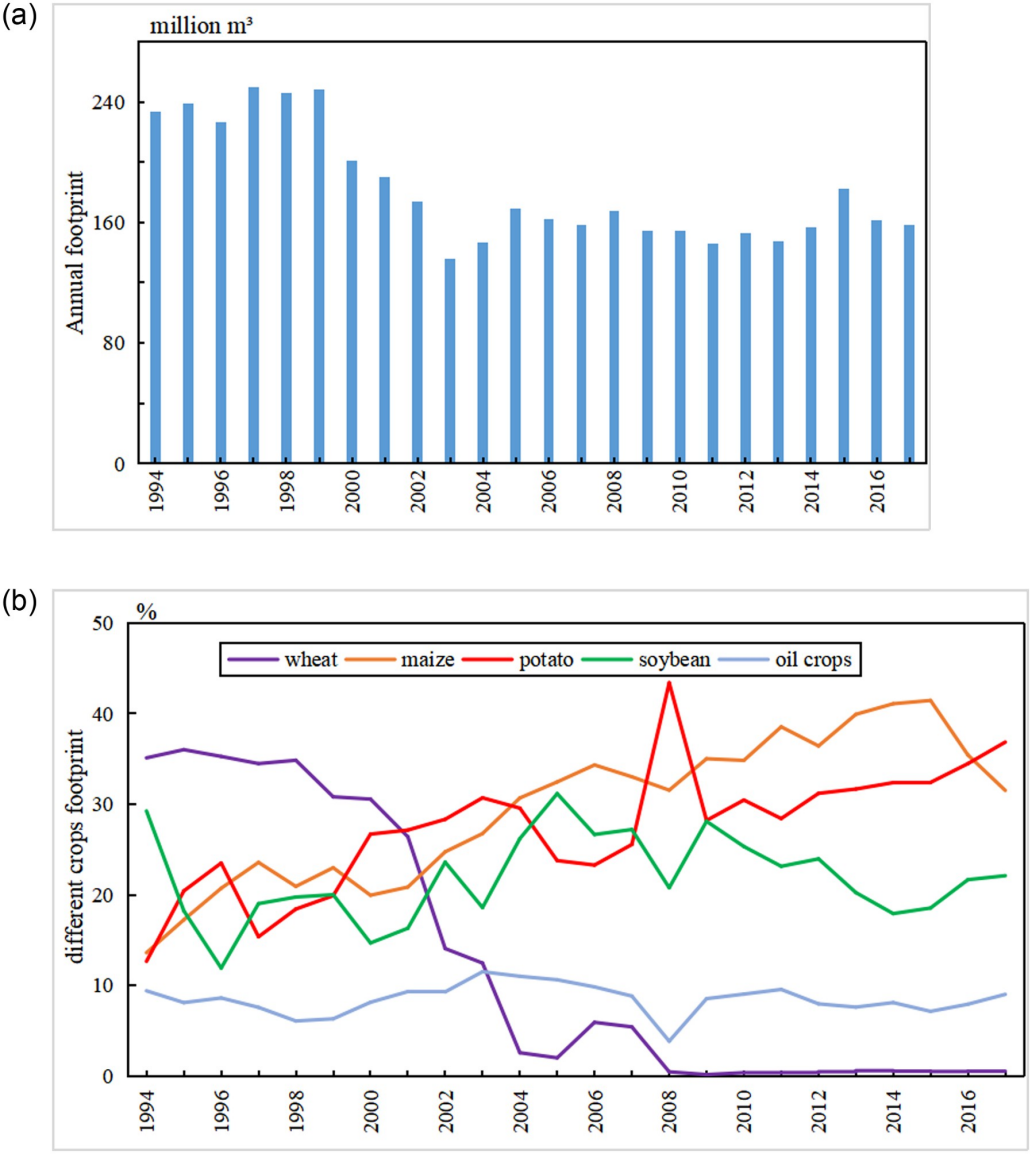
Agricultural footprint.

This study calculated crop WF during the period of 1994–2017 because of crop output was inaccessible before 1994 (Fig 9). The agricultural WF in the Yanhe River Basin showed a rapid downward trend, with the highest value of 250 million m^3^ in 1997 and the lowest value of 136 million m^3^ in 2003. The average annual WF of agricultural products was approximately 160 million m^3^, with annual decreases of approximately 4.07 million m^3^/yr.

The WF of each crop in the Yanhe River Basin changed greatly during the study period. Wheat was no longer planted in the Baota District after 2005 and in Ansai County after 2008, and the yield in Yanchang County decreased from 16238 tons in 1994 to 353 tons in 2017. Therefore, wheat WF decreased from 81.97 million m^3^ in 1994 to 0.84 million m^3^ in 2017, and the percentage ranged from 35.1 to 0.52. The WF proportions of corn and potato both had obvious upward trends, and the corn WF increased from 31.75 million m^3^ to 50.58 million m^3^; the proportion increases from 13.6 percent to 31.5 percent. The potato WF increased from 29.52 million m^3^ to 59.13 million m^3^, and the proportion increased from 12.65 percent to 36.85 percent. Soybean and oil crop WFs fluctuated in different years, but overall change trends were not obvious during the study period. Winter wheat WF was the largest and far more than the other crop WFs, consequently, the sum of the total crop WFs decrease during 1994–2017 although all crop WFs except wheat WF increased.

#### Ecological WF

The vegetation WF was calculated according to Eqs (8) and (9) in ArcGIS by vegetation type, soil texture and potential evaporation, and the results are presented in Table 5. The forest WF was larger in May, June and July because the actual evapotranspiration rates of these three months were greater than those of the other months, and the minimum value appeared in October because leaves begin to wither and fall, and the water demand is the smallest in the growth stage. The total forest WF increased from 5.22 billion m^3^ to 19.58 billion m^3^ during 1980–2017. After 2000, the forest WF began to increase rapidly, and the maximum value appeared in 2017.

**Table 5:** WFs of the forest from April to October in different years (billion m3).

Month	1980	1995	2000	2005	2010	2017
4	0.74	0.81	0.91	1.32	2.52	3.35
5	0.85	1.04	1.17	1.40	3.02	4.03
6	0.95	0.96	0.89	1.52	3.31	3.74
7	0.86	0.85	0.99	1.30	2.71	3.56
8	0.74	0.56	0.67	0.93	2.05	2.31
9	0.62	0.43	0.50	0.69	1.48	1.73
10	0.46	0.27	0.24	0.45	1.05	0.87
Summary	5.22	4.92	5.37	7.6	16.14	19.58

The grass WF was larger in May and June but smallest in October in the grass growth stage, which is the same as the monthly characteristics of forests. Table 1 demonstrates that grassland area decreased from 3565.2 km^2^ to 3547.09 km^2^, which indicates that GGP had comparatively little effect on grassland, so the grass WF had no obvious change and decreased slightly from 18.71 billion m^3^ to 15.44 billion m^3^ (Table 6).

**Table 6:** WFs of grassland from April to October in different years (billion m3).

Month	1980	1995	2000	2005	2010	2017
4	2.66	2.33	3.04	3.33	2.43	2.64
5	3.04	3.15	3.86	3.55	2.92	3.18
6	3.41	3.41	2.97	3.85	3.21	2.95
7	3.09	3.20	3.23	3.15	2.63	2.81
8	2.66	2.80	2.21	2.31	1.98	1.82
9	2.21	1.98	1.63	1.66	1.43	1.37
10	1.64	1.38	0.73	1.04	1.02	0.68
Summary	18.71	18.26	17.67	18.90	15.61	15.44

#### Agricultural virtual water flow

**Fig 10:**
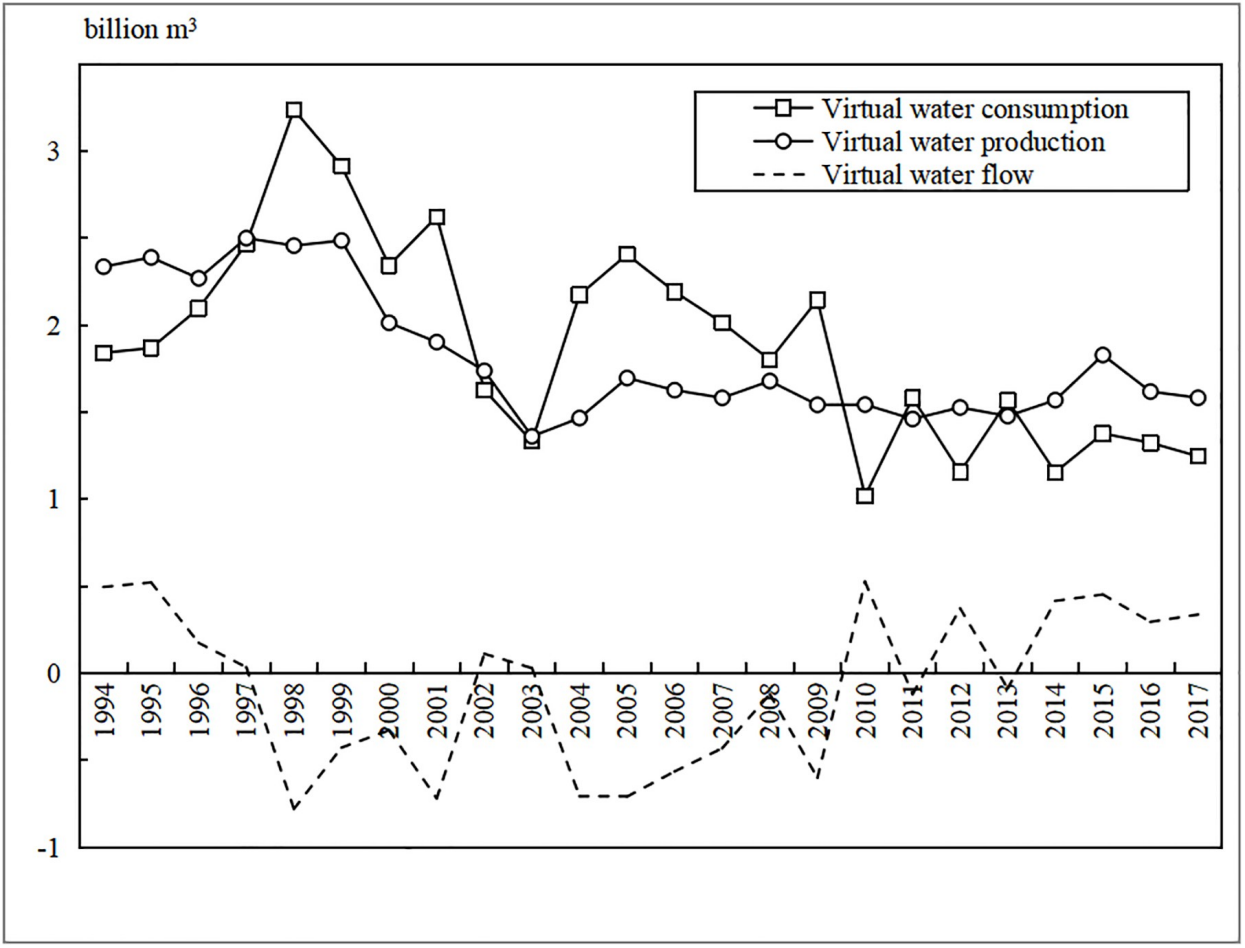
Agricultural virtual flows during 1994–2017.

The population in the Yanhe River basin increases from 60.92 to 79.83 ten thousand, but per capita grain consumption decreased from 208.04 kg to 108.5 kg, and the regional total grain consumption WF had a downward trend during 1994–2017. Crop yields varied greatly from year to year, leading to an irregular decrease in grain production WF. Fig 10 indicates that from 1994 to 2017, the grain consumption WF in the Yanhe River Basin ranged from 1.01 billion m^3^ to 3.24 billion m^3^, and the grain production WF ranged from 1.36 billion m^3^ to 2.5 billion m^3^. The grain production WF before 2000 was more than 2 billion m^3^ and was approximately 1.5 billion m^3^ after 2003. In 1994–1997, 2002–2003, 2012 and 2014–2017, virtual water flowed from the Yanhe River basin to the outside. From 1994 to 2017, approximately 8 million m^3^ of virtual water came from outside the region annually. Therefore, virtual grain water in the Yanhe River Basin imported from the outside, which can alleviate the pressure of water resources in the basin to a certain extent.

### EWSI

The EWSI was calculated for 1995, 2000, 2005, 2010 and 2017, and the results are shown in Table 7. The total WF showed an increasing trend due to the significant increase in forest WF, although agricultural and grass WFs showed decreasing trends during the study years. However, the ecological water stress index had no obvious temporal change because the change trend of generalized water resources was the same as that of the regional total WF. The EWSI was less than 1 in the five studied years in both land use scenarios from the point view of generalized water resources. The ESWI in scenario II was larger than that in scenario I in the five years, and this demonstrates that the GGP slightly increased regional water stress because the difference in ESWI is approximately 0.01 between the two land use scenarios.

**Table 7:** Ecological water stress index values of the two land use scenarios in the Yanhe River Basin.

Year	WF (billion m^3^)	Scenario I		Scenario II		Scenario II -I
		Generalized water resources (billion m^3^)	ESWI	Generalized water resources (billion m^3^)	ESWI	ESWI
**1995**	25.2	35.45	0.711	34.50	0.730	0.02
**2000**	26.32	31.75	0.829	31.31	0.841	0.01
**2005**	31.26	40.08	0.780	39.79	0.786	0.01
**2010**	36.12	42.88	0.842	42.51	0.850	0.01
**2017**	35.78	46.10	0.776	45.66	0.784	0.01

## Discussion

### Model calibration and uncertainty programs

Table 4 and Fig 4 indicated a good SWAT model performance for streamflow simulation in the Yanhe River basin. However, factors such as parameter selection, rainstorm, observation data missing and heterogeneity within HRUs can all lead to deviation of simulated data from observed data.

Currently, SWAT Calibration and Uncertainty Programs (SWAT-CUP) is mostly to determine the sensitive parameters and the optimal value. CN2 is mostly recognized as the most important parameter in SWAT streamflow simulations [61, 62] because SWAT uses the soil conservation service streamflow curve method to calculate the surface streamflow. In addition, the surface streamflow in most watersheds plays a dominant role in the total annual streamflow. Therefore, CN2 plays important role in streamflow simulation and it is critical for model accuracy. Previous research showed that there was a great difference in parameter of CN2 in different study in Yanhe river basin (e.g. -0.481, Ganguyi station, Gong JF [63]. 32, Ganguyi station, Zhu Y [64]. 0.14, Ganguyi station, Lian QH [65]. 0.15, Ganguyi station, Zhao CP [66]. -0.48, Xinghe station, -1.5, Ansai station, -1.61, Zaoyuan station, -1.16, Yan’an station, Zhang HB [67]). The CN2 values of main land use types were 78.03 (AGRL), 62.31 (FRST) and 70 (PAST), respectively in this paper. The results were consistent with other regions on the Loess Plateau (i.e. 64.4–85.7, Shi WH [68], the Loess Plateau. 73.3–89.8, Feng J [69], Anjiagou basin and Longtan basin in Dingxi city in Gansu province. 71.12–76.72, Li CB [70]. Anjiagou basin in Dingxi city in Gansu province. 70–80, Wang HY [71], Caijiachuan basin in Jixian city in Shanxi province. 64.47–73.88, Deng JC [72], Yangou basin in Yan’an in Shaanxi province. 76.61, Xu Z [73], Jiuyuangou basin in Suide city in Shaanxi province. 40–98, Xu Z [74], Middle reaches of the Yellow River). Different sub-basin and slope division, land use precision, and meteorological stations may lead to different parameter values. There was difference for optimal CN2 in SWAT-CUP among different paper and the CN2 values for different land use types in many papers were similar.

Studies have shown that multiple outlets in SWAT model simulation perform better than a single outlet [75, 76]. Ganguyi hydrology station was a single calibration outlet in this paper that may be one of the reasons for relative low accuracy. Simulation accuracy of areas for severe land use changes is smaller than that of areas with gentle land use change because land use cannot be input into the model every year. For example, simulation results in Xihe River basin [77] and Qingshuihe basin [78] were obviously better than that in the Loess Plateau, The upper reaches of the simulation results was better than the middle and lower reaches in the Yellow River basin [79].

### Assessment of the water balance components and uncertainty analysis

This study investigated the spatial-temporal impact of the GGP on regional water resources and ecological water stress in the Yanhe River Basin in 1980 and 2017 using the results of the SWAT model. Many researchers have explored blue water and green water resources since the appearance of the concept using different hydrological cycle models [43]. Rost et al. [80] used the Lund-Potsdam-Jena managed land (LPJmL) model to quantify the global consumption of both blue and green water in 1971–2000. SWAT is currently the most commonly used model to simulate the effects of land-use change and climate change on the hydrological cycle. Many previous studies have demonstrated that the results of SWAT simulations are reliable when exploring land-use and climate change effects on the hydrological cycle in many river basins around the world [81, 82].

The most important elements of the water balance for one watershed include precipitation, SURQ, LATQ, GWQ, ET and SW [83]. In this study, hydrological components other than precipitation were analyzed to investigate hydrological cycle changes caused by the GGP. Average annual water balance components simulated by the SWAT model is presented in Table 8. This result shows that SRUQ decreased, ET increases were accompanied by vegetation cover and urban land use increases in the Yanhe River basin, which is consistent with the same phenomenon found in many regions around the world [84–86]. ET was the largest contributor to water loss in this region. There was only an 8.49 mm difference in water yield between Scenario II and Scenario I because SURQ decreased while GWQ and LATQ increased after the GGP. The drastic interannual changes of water yield in both scenarios may be caused by parameters other than land use change.

**Table 8:** Average annual water balance components.

Water Balance Component	Depth(mm)	
	Scenario I	Scenario II
SRUQ	65.07	47.51
LATQ	55.06	57.55
GWQ	70.04	76.62
ET	301.98	314.26
SW	81.99	73.09
Water yield	190.17	181.68

Water yield = SURQ + GWQ + LATQ − T_loss_. Multiyearly T_loss_ is always considered as 0.

In this study, the accuracy of SWAT simulation was one of the important key factors because many results, such as blue water, green water, and hydrological elements (green water flow, green water storage, SURQ, GWQ and LATQ), were all outputs of the model. The R^2^ and NSE values in this study were within the accuracy requirement of model establishment, and the accuracy was close to the results of other studies in this river basin [87–89]. During the SWAT simulation, land use was classified into six types that cannot actually represent the land use type, and the land use module should be improved. Data from four meteorological stations, Yanchang, Ansai, Zhidan and Yanan, were input into SWAT, and the precipitation data was also from meteorological stations. More meteorological and precipitation data may enhance the simulation accuracy. Reservoir information, such as Wangyao, can be used in the model to consider human impact, while improving DEM data can also improve simulation accuracy [90].

Only five crops, wheat, summer maize, potato, soybean and oil crops, were considered, while other agricultural WFs were not included because of the lower yield in the Yanhe River Basin; therefore, the agricultural WF was less than the actual total agricultural WF in the region. The most important varieties of trees in the Loess Plateau are robin pseudoacacia, Platycladus orientalis, Pinus tabulaeformis and so on. Different experiments on forests on the Loess Plateau show that the ecological water demand coefficient of arbor forests is 0.757 and that of shrub forests is 0.612 [91]; the water demand coefficient of forestland is 0.76, that of shrub forests is 0.61 and that of sparse forestland is 0.48 [92, 93]. There are no precise and uniform water demand coefficients of forest and grass on the Loess Plateau because there have been few experimental studies on it. This paper cites the existing study results and does not distinguish forest types because of a lack of experiments and image interpretation accuracy. The above factors will all influence the ecological WF and EWSI.

### Implication of the GGP on water resources system

**Fig 11:**
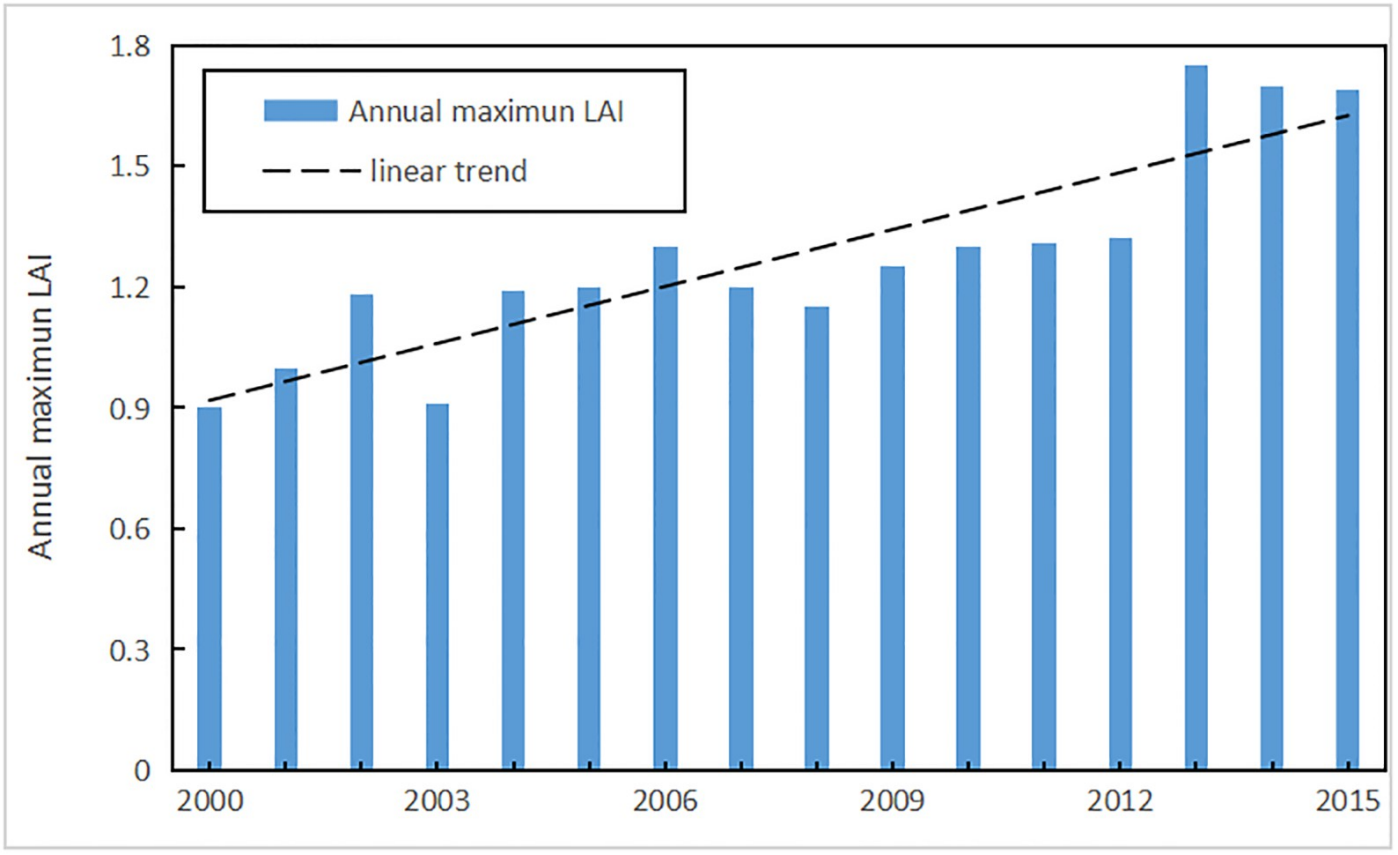
Change trend of the annual maximum LAI for the Yanhe River Basin during 2000–2015.

The results of this study show that blue water resources decreased and green water increased obviously, but generalized water resources decreased slightly; SURQ and SW decreased but ET increased significantly over the whole basin with the achievements of the GGP; these results are similar to those of Yang [87] and Yang [94] in the Yanhe River Basin. Large amounts of cultivated lands were transformed into forestlands, reducing slope surface runoff and ultimately improved green water storage, which is important for restoring vegetation within this region [95]. The reason that the blue water decreased and green water increased may be the shift from the former to the latter. With the increase in vegetation coverage in the Yanhe River Basin, the accumulation of bark debris and leaf litter significantly increased soil surface roughness, slowing the runoff rate, increasing infiltration and intensifying evaporation [96]. Fig 11 shows that the annual maximum leaf area index (LAI) increased from 0.9 to 1.75 during 2000–2014. The annual maximum LAI had an increasing trend since 2000, and a larger LAI can increase the interception capacity and rain loss. Water evaporating before infiltration, increases green water flow and reduces surface runoff.

The water demand of increasing vegetation in the semiarid climate of the Loess Plateau has reduced the soil water content of the top 3–5 m to almost withering humidity and further exacerbated soil drying [97]. The ET in the growing season limits vegetation growth [98]. Water resources cannot satisfy water requirements due to forest and grass growth leading to slow growth of trees and low yields of forests because precipitation in this region did not significantly increase during the study period although evaporation increased. The Chinese government plans to invest another US$9.5 billion in the GGP on the Loess Plateau by 2050 [99]. The results of this study show that although this project has contributed to increasing forestland area and green water resources, this has been at the cost of detectable reductions in blue water resources, especially in river runoff. Studies have indicated that current vegetation productivity in the Loess Plateau is already close to net primary productivity (NPP), and total water resources will inevitably decrease for human use to less than the required amount if vegetation coverage continues to increase [22]. Therefore, future GGPs should consider water stress and other negative impacts.

### GGP sustainability

Natural rainfall data from 43 articles and 331 runoff experimental graphics in the Loess Plateau have been integrated and synthesized to analyze the land use change impact on runoff, and the results show that artificially promoted vegetation has a substantial negative impact on runoff formation [100]. Blue water decreased in all of the studied years, and the difference between the two land use scenarios is -72.08 million m^3^. Vegetation cover increases from 4347.6 km^2^ to 6565.61 km^2^. SURQ decline reduced the amount of water resources that people can use directly; hence, during the implementation of the GGP more detailed investigation about how to adjust vegetation types according to regional precipitation, topographical conditions and temperature should be carried out. The average green water increase indicates that the ecological environment has improved significantly, and the EWSI became slightly worse after the GGP. Therefore, vegetation restoration has both positive and negative impacts on water resources and ecological systems. Appropriate trees or other vegetation types should be planted to maintain the balance between ecosystems and water resource systems in the future.

Compared with the previous research on impact of land use change on regional water resources, this paper evaluates the role of green water resources in the basin; analyze regional water stress variation adopting the concept of generalized water resources. Taking green water as one part of regional water resources and taking forest and grass land water footprint as one part of water requirement, this method can discuss whether vegetation restoration aggravate or alleviate regional water resource stress more comparatively.

## Conclusions

The concern about the impact of vegetation restoration on regional water resources has increased globally in recent years, and it is necessary to integrate the SWAT model and WF to explore the problem. This paper presents a framework to analyze the spatial and temporal characteristics of blue water, green water and each hydrological element as well as EWSI changes. The Yanhe River basin is taken as a case study to explore the negative and positive impacts of the GGP on regional water resources. The results show that the vegetation cover area increased significantly since the implementation of the GGP, blue water, and SURQ decreased substantially, which is consistent with the results of many previous investigations. Green water and generalized water resources had increasing and decreasing trends, respectively, which were caused by land use change during 1980–2017. The EWSI became slightly worse from the perspective of the generalized water resources and the WF concept.

## Supporting information

S1 Data (XLSX)

S1 File (VSD)

## References

[1] Wang S, Fu BJ, Piao SL, Lü YH, Ciais P, Feng XM, et al. Reduced sediment transport in the Yellow River due to anthropogenic changes. Nature Geoscience. 2016; 9: 38–41. doi: 10.1038/ngeo2602

[2] Zheng HY, Miao CY, Zhang GH, Li XY, Wang S, Wu JW, et al. Is the runoff coefficient increasing or decreasing after ecological restoration on China’s Loess Plateau. International Soil and Water Conservation Research. 2021; 9(3): 333–343. doi: 10.1016/j.iswcr.2021.04.009

[3] Deng L, Shangguan ZP, Li R. Effects of the grain-for-green program on soil erosion in China. International Journal of Sediment Research. 2012; 27(1): 120–127. doi: 10.1016/S1001-6279(12)60021-3

[4] Qiu LJ, Chen YT, Wu YP, Xue QY, Shi ZY, Lei XH, et al. The Water Availability on the Chinese Loess Plateau since the Implementation of the Grain for Green Project as Indicated by the Evaporative Stress Index. Remote Sensing. 2021; 13(16): 3302. doi: 10.3390/rs13163302

[5] Zhang DJ, Ge WY, Zhang Y. Evaluating the vegetation restoration sustainability of ecological projects: A case study of Wuqi County in China. Journal of Cleaner Production. 2020; 264: 121751. doi: 10.1016/j.jclepro.2020.121751

[6] Zhang F, Xing ZS, Rees HW, Dong YL, Li S, Meng FR. Assessment of effects of two runoff control engineering practices on soil water and plant growth for afforestation in a semi-arid area after 10 years. Ecological Engineering. 2014; 64: 430–442. doi: 10.1016/j.ecoleng.2013.12.024

[7] Zhang SL, Yang DW, Yang HB, Lei HM. Analysis of the dominant causes for runoff reduction in five major basins over China during 1960–2010. Advances in Water Science. 2015; 26(5): 605–613. doi: 10.14042/j.cnki.32.1309.2015.05.001

[8] Li S, Liang W, Fu BJ, Lü YH, Fu SY, Wang S, et al. Vegetation changes in recent large-scale ecological restoration projects and subsequent impact on water resources in China’s Loess Plateau. Science of Total Environment. 2016; 569–570: 1032–1039. doi: 10.1016/j.scitotenv.2016.06.141 27387800

[9] Chen LD, Wei W, Fu BJ, Lv YH. Soil and water conservation on the Loess Plateau in China: review and perspective. Progress in Physical Geography. 2007; 31(4): 389–403. doi: 10.1177/0309133307081290

[10] Liu W, Shi CX, Zhou YY. Trends and attribution of runoff changes in the upper and middle reaches of the Yellow River in China. Journal of Hydro-Environment Research. 2021; 37: 57–66. doi: 10.1016/j.jher.2021.05.002

[11] Zhang SL, Yang DW, Yang YT, Piao SL, Yang HB, Lei HM, et al. Excessive afforestation and soil drying on China’s Loess Plateau. Journal of Geophysical Research: Biogeosciences. 2018; 123: 923–935. doi: 10.1002/2017JG004038

[12] Wen X, Deng XZ, Zhang F. Scale effects of vegetation restoration on soil and water conservation in a semi-arid region in China: Resources conservation and sustainable management. Resources Conservation and Recycling. 2019; 151: 104474. doi: 10.1016/j.resconrec.2019.104474

[13] Chen H, Fleskens L, Baartman J, Wang F, Moolenaar S, Ritsema C. Impacts of land use change and climatic effects on streamflow in the Chinese Loess Plateau: A meta-analysis. Science of Total Environment. 2020; 703: 134989. doi: 10.1016/j.scitotenv.2019.134989 31734503

[14] Fashey B, Jackson R. Hydrological impacts of converting native forests and grasslands to pine plantations, South Island, New Zealand. Agriculture and Forest Meteorology. 1997; 84(1–2): 69–82. doi: 10.1016/S0168-1923(96)02376-3

[15] Hosseini M, Ghafouri A, Amin M, Tabatabaei M, Goodarzi M, Kolahchi AA. Effects of land use changes on water balance in Taleghan Catchment, Iran. Journal of Agriculture Science and Technology. 2012; 14(5): 1159–1172. doi: 10.1111/j.1744-697X.2012.00258.x

[16] Jia XX, Zhao CL, Wang YQ, Zhu YJ, Wei XR, Shao MA. Traditional dry soil layer index method overestimates soil desiccation severity following conversion of cropland into forest and grassland on China’s Loess Plateau. Agriculture, Ecosystems and Environment. 2020; 291: 106794. doi: 10.1016/j.agee.2019.106794

[17] Liu Y, Miao HT, Huang Z, Cui Z, He HH, Zheng JY, et al. Soil water depletion patterns of artificial forest species and ages on the Loess Plateau (China). Forest Ecology and Management. 2018; 417: 137–143. doi: 10.1016/j.foreco.2018.03.005

[18] Ge J, Pitman AJ, Guo WD, Zan BL, Fu CB. Impact of revegetation of the Loess Plateau of China on the regional growing season water balance. Hydrology and Earth System Sciences Discussion. 2020; 24(2): 515–533. doi: 10.5194/hess-24-515-2020

[19] Wang ZY, Peng DL, Xu DY, Zhang XY, Zhang Y. Assessing the water footprint of afforestation in Inner Mongolia, China. Journal of Arid Environment. 2020; 182: 104257. doi: 10.1016/j.jaridenv.2020.104257

[20] Falkenmark M. The New Blue and Green Water Paradigm: Breaking New Ground for Water Resources Planning and Management. Journal of Water Resources Planning and Management. 2006; 132(3): 129–132. doi: 10.1061/(ASCE)0733-9496(2006)132:3(129)

[21] Jian SQ, Zhao CY, Fang SM, Yu K. Effects of different vegetation restoration on soil water storage and water balance in the Chinese Loess Plateau. Agriculture and Forest Meteorology. 2015; 206: 85–96. doi: 10.1016/j.agrformet.2015.03.009

[22] Feng XM, Fu BJ, Piao SL, Wang S, Ciais P, Zeng ZZ, et al. Revegetation in China’s Loess Plateau is approaching sustainable water resource limits. Nature Climate Change. 2016; 6: 1019–1022. HYPERLINK "https://www.nature.com/articles/nclimate3092/" doi: 10.1038/nclimate3092

[23] Zhao JM, Chen CH, Li J. Impacts of soil and water conservation on water resources carrying capacity in Yellow River basin. Journal of Hydraulic Engineering. 2010; 41(9): 1079–1086. doi: 10.13243/j.cnki.slxb.2010.09.007

[24] Hoekstra A, Hung PQ. Virtual Water Trade: A quantification of virtual water flows between nations in relation to international crop trade. Water Science and Technology. 2002; 49(11): 203–209.

[25] Joseph N, Ryu D, Malano HM, George B, Sudheer KP. A review of the assessment of sustainable water use at continental-to-global scale. Sustainable Water Resources Management. 2020; 6(2): 18. doi: 10.1007/s40899-020-00379-7

[26] Mekonnen MM, Hoekstra AY. The green, blue and grey water footprint of crops and derived crop products. Hydrology and Earth System Sciences. 2011; 8(1): 763–809. doi: 10.5194/hessd-8-763-2011

[27] Jeyrani F, Morid S, Srinivasan R. Assessing basin blue–green available water components under different management and climate scenarios using SWAT. Agriculture Water Management. 2021; 256: 107074. doi: 10.1016/j.agwat.2021.107074

[28] Zhao YZ, Mu XM, Yan BW, Zhao GJ. Influence of vegetation restoration on runoff and sediment of Yanhe basin. Journal of Sediment Research. 2014; 4: 67–73. doi: 10.16239/j.cnki.0468-155x.2014.04.007

[29] Zhang J, Chen HS, Fu ZY, Wang KL. Effects of vegetation restoration on soil properties along an elevation gradient in the karst region of southwest China. Agriculture, Ecosystems and Environment. 2021; 320: 107572. doi: 10.1016/j.agee.2021.107572

[30] Li Q, Shi XY, Wu QQ. Exploring suitable topographical factor conditions for vegetation growth in Wanhuigou catchment on the Loess Plateau, China: A new perspective for ecological protection and restoration. Ecological Engineering. 2020; 158: 106053. doi: 10.1016/j.ecoleng.2020.106053

[31] Zhou ZX, Li J. The correlation analysis on the landscape pattern index and hydrological processes in the Yanhe watershed, China. Journal of Hydrology. 2015; 524: 417–426. doi: 10.1016/j.jhydrol.2015.02.028

[32] Bieger K, Arnold J, Rathjens H, White MJ, Bosch DD, Allen PM, et al. Introduction to SWAT+, A Completely Restructured Version of the Soil and Water Assessment Tool. Journal of American Water Resources Association. 2017; 53(1): 115–130. doi: 10.1111/1752-1688.12482

[33] Setegn SG, Srinivasan R, Dargahi B, Melesse AM. Spatial delineation of soil erosion vulnerability in the Lake Tana Basin, Ethiopia. Hydrological Processes. 2009; 23(26): 3738–3750. doi: 10.1002/hyp.7476

[34] Luan XB, Wu PT, Sun SK, Wang YB, Gao XR. Quantitative study of the crop production water footprint using the SWAT model. Ecological Indicators. 2018; 89: 1–10. doi: 10.1016/j.ecolind.2018.01.046

[35] Cibin R, Chaubey I, Engel B. Simulated watershed scale impacts of corn stover removal for biofuel on hydrology and water quality. Hydrological Processes. 2012; 26(11): 1629–1641. doi: 10.1002/hyp.8280

[36] Giri S, Nejadhashemi AP, Woznicki S, Zhang Z. Analysis of best management practice effectiveness and spatiotemporal variability based on different targeting strategies. Hydrological Processes. 2014; 28(3): 431–445. doi: 10.1002/hyp.9577

[37] Veettil AV, Mishra AK. Water security assessment using blue and green water footprint concepts. Journal of Hydrology. 2016; 542: 589–602. doi: 10.1016/j.jhydrol.2016.09.032

[38] Zhao AZ, Zhu XF, Liu XF, Pan YZ, Zuo DP. Impacts of land use change and climate variability on green and blue water resources in the Weihe River Basin of northwest China. Catena. 2016; 137: 318–327. doi: 10.1016/j.catena.2015.09.018

[39] Shrestha NK, Du XZ, Wang JY. Assessing climate change impacts on fresh water resources of the Athabasca River Basin, Canada. Science of Total Environment. 2017; 601–602: 425–440. doi: 10.1016/j.scitotenv.2017.05.013 28570976

[40] Abbaspour KC. SWAT Calibration and Uncertainty Programs. A User Manual Swiss Federal Institute of Aquatic Science and Technology, Switzerland, 2015.

[41] Wang QR, Liu RM, Men C, Guo LJ, Miao YX. Temporal-spatial analysis of water environmental capacity based on the couple of SWAT model and differential evolution algorithm. Journal of Hydrology. 2019; 569: 155–166. doi: 10.1016/j.jhydrol.2018.12.003

[42] Phiri WK, Vanzo D, Banda K, Nyirenda E, Nyambe IA. A pseudo-reservoir concept in SWAT model for the simulation of an alluvial floodplain in a complex tropical river system. Journal of Hydrology: Regional Studies. 2021; 33: 100770. doi: 10.1016/j.ejrh.2020.100770

[43] Liang J, Liu Q, Zhang H, Li XD, Qian Z, Lei MQ, et al. Interactive effects of climate variability and human activities on blue and green water scarcity in rapidly developing watershed. Journal of Cleaner Production. 2020; 265: 121834. doi: 10.1016/j.jclepro.2020.121834

[44] Veettil AV, Mishra AK. Potential influence of climate and anthropogenic variables on water security using blue and green water scarcity, Falkenmark index, and freshwater provision indicator. Journal of Environment and Management. 2018; 228: 346–362. doi: 10.1016/j.jenvman.2018.09.012 30241040

[45] Liu JG, Zehnder AJB, Yang H. Global consumptive water use for crop production: the importance of green water and virtual water. Water Resources Research. 2009; 45(5): W05428. doi: 10.1029/2007WR006051

[46] Zang CF, Liu JG. Trend analysis for the flows of green and blue water in the Heihe River basin, northwestern China. Journal of Hydrology. 2013; 502: 27–36. doi: 10.1016/j.jhydrol.2013.08.022

[47] Sun SK, Wang YB, Liu J, Wu PT. Quantification and evaluation of water footprint of major grain crops in China. Journal of Hydraulic Engineering. 2016; 47(9): 1115–1124. doi: 10.13243/j.cnki.slxb.20150956

[48] Novoa V, Ahumada-Rudolph R, Rojas O, Sáez K, Barrera F, Arumí JL. Understanding agricultural water footprint variability to improve water management in Chile. Sciences of the Total Environment. 2019; 670: 188–199. doi: 10.1016/j.scitotenv.2019.03.127 30903892

[49] Wu PT, Sun SK, Wang YB, Li XL, Zhao XN. Research on the quantification methods for water footprint of crop production. Journal of Hydraulic Engineering. 2017; 48(6): 651–660+669. doi: 10.13243/j.cnki.slxb.20160740

[50] Lin LX, Gao XR, Zhao Y, Wang LZ, An TL, Liu C, et al. Evaluation of the water consumption of animal products and the virtual water flow pattern associated with interprovincial trade in China. Journal of Cleaner Production. 2021; 328: 129599. doi: 10.1016/j.jclepro.2021.129599

[51] Chi DK, Wang H, Li XB, Liu HH, Li XH. Estimation of the ecological water requirement for natural vegetation in the Ergune River basin in Northeastern China from 2001 to 2014. Ecological Indicators. 2018; 92: 141–150. doi: 10.1016/j.ecolind.2017.04.014

[52] Zhao F, Li H, Lia CH, Cai YP, Wang X, Liu Q. Analyzing the influence of landscape pattern change on ecological water requirements in an arid/semiarid region of China. Journal of Hydrology. 2019; 578: 124098. doi: 10.1016/j.jhydrol.2019.124098

[53] Min QW, He YT, Li WH, Li GC. Estimation of forests’ ecological water requirement based on agrometeorology: taking Jinghe watershed as an example. Acta Ecological Sinica. 2004; 24(10): 2130–2135.

[54] Li JY, Zhang WJ. Ecological water requirement by forest and grass in central and southern Ningxia hui autonomous region. Bulletin of soil and water conservation. 2014; 34(2): 276–280. doi: 10.13961/j.cnki.stbctb.2014.02.057

[55] Raskin P, Gleick P, Kirshen P, Pontius G, Strzepek K. Water Futures: Assessment of Long-range Patterns and Prospects. Stockholm, Sweden: Stockholm Environment Institute. 1997.

[56] Oki T, Kanae S. Global hydrological cycles and world water resources. Science. 2006; 313(5790): 1068–1072. doi: 10.1126/science.1128845 16931749

[57] Kaewmai R, Grant T, Eady S, Mungkalasiri J, Musikavong C. Improving regional water scarcity footprint characterization factors of an available water remaining (AWARE) method. Science of the Total Environment. 2019; 681: 444–455. doi: 10.1016/j.scitotenv.2019.05.013 31128340

[58] Nilsalab P, Gheewala SH, Silalertruksa T. Methodology development for including environmental water requirement in the water stress index considering the case of Thailand. Journal of Cleaner Production. 2017; 167: 1002–1008. doi: 10.1016/j.jclepro.2016.11.130

[59] Athira RP. Calibration of hydrological models considering process interdependence: A case study of SWAT model. Environment Modelling and Software. 2021; 144: 105131. doi: 10.1016/j.envsoft.2021.105131

[60] Moriasi DN, Arnold JG, Van Liew MW, Bingner RL, Harmel RD, Veith TL. Model evaluation guidelines for systematic quantification of accuracy in watershed simulations. Transaction of the Asabe. 2007; 50(3): 885–900. doi: 10.13031/2013.23153

[61] Li M, Di ZH, Duan QY. Effect of sensitivity analysis on parameter optimization: Case study based on streamflow simulations using the SWAT model in China. Journal of Hydrology. 2021; 603: 126896. doi: 10.1016/j.jhydrol.2021.126896

[62] Sharma A, Patel PL, Sharma PJ. Influence of climate and land-use changes on the sensitivity of SWAT model parameters and water availability in a semi-arid river basin. Catena. 2022; 215: 106298. doi: 10.1016/j.catena.2022.106298

[63] Gong JF, Li ZB, Li P, Ren ZP, Yang YY, Han L, et al. Spatial distribution of runoff erosion power based on SWAT Model in Yanhe River Basin. Transactions of the Chinese Society of Agricultural Engineering. 2017; 33(13): 120–126. doi: 10.11975/j.issn.1002-6819.2017.13.016

[64] Zhu Y, Fang XQ, Wang K, Zhu QA. Simulation of Monthly Runoff in the Yanhe River Basin Using SWAT Model. Journal of Yangtze River Scientific Research Institute. 2016; 33(10): 41–45. doi: 10.11988/ckyyb.20150681

[65] Lian QH. Runoff and sediment variation in the Yanhe watershed and their response to land use cover Change. M.Sc. Thesis, Northwest A & F University. 2020. http://cdmd.cnki.com.cn/Article/CDMD-10712-1020907469.htm

[66] Zhao CP. Runoff Response to Land Use Change in the Yan River Using SWAT Model. M.Sc. Thesis, University of Chinese Academy of Sciences. 2015. http://cdmd.cnki.com.cn/Article/CDMD-80129-1015996169.htm

[67] Zhang HB, Zhi T, Wei XC, Dang CH, Xia Y, Gao WB. Simulation of runoff process in the middle reaches of the Yellow River based on SWAT-MODFLOW model and its response to the greening of the loess plateau. Journal of North China university of water resources and electric power (natural science edition). 2020; 41(6): 1–10. doi: 10.19760/j.ncwu.zk.2020069

[68] Shi WH, Wang N, Wang MM, Li DH. Revised runoff curve number for runoff prediction in the Loess Plateau of China. Hydrological Processes. 2021; 35: e14390. doi: 10.1002/hyp.14390

[69] Feng J, Wei W, Feng QY. The Runoff curve number of SCS-CN moethod in s loess hilly region. Acta Ecologica Sinica. 2021; 41(10): 4170–4181. doi: 10.5846/stxb201912082665

[70] Li CB, Qin JW, Li JB. Application of computational curve number to precipitation-runoff simulation in a typical Watershed in Chinese Loess Plateau. Journal of Arid Land Resources and Environment. 2008; 8: 67–70. doi: 10.13448/j.cnki.jalre.2008.08.025

[71] Wang HY, Zhang ZQ, Zha TG, Zhu YS, Zhang JJ, Zhu JZ. Modification of SCS-CN model for estimating event rainfall runoff for small watersheds in the Loess Plateau, China. Journal of Beijing Forestry University. 2016; 38(08): 71–79. doi: 10.13332/j.1000-1522.20150508

[72] Deng JC, Gao P, Mu XM, Zhao GJ, Sun WY, Tian P, et al. Study on Calculation Method of Runoff Curve Number of SCS-CN Model in Loess Area Under Simulated Rainfall. Yellow River. 2018; 40(4): 9–14+18. doi: 10.3969/j.issn.1000-1379.2018.04.003

[73] Xu Z, Wu L, Wu YQ, Xu RR. Improvement and runoff prediction of SCS-CN model. Hydro-Science and Engineering. 2018; 3: 32–39. doi: 10.16198/j.cnki.1009-640X.2018.03.005

[74] Xu Z, Zhang SH, Yang XY. Water and sediment yield response to extreme rainfall events in a complex large river basin: A case study of the Yellow River Basin, China. Journal of Hydrology. 2021; 597(2): 126183. doi: 10.1016/j.jhydrol.2021.126183

[75] Hu J, Ma J, Nie C, Xue LQ, Zhang T, Ni FQ, et al. Attribution Analysis of Runoff Change in Min-Tuo River Basin based on SWAT model simulations, China. Scientific Reports. 2020; 10: 2900. doi: 10.1038/s41598-020-59659-z 32075996 PMC7031534

[76] Wang H, Sun FB, Xia J, Liu WB. Impact of LUCC on streamflow based on the SWAT model over the Wei River basin on the Loess Plateau in China. Hydrology and Earth System Sciences. 2017; 21(4): 1929–1945. doi: 10.5194/hess-21-1929-2017

[77] Guo SS, Zhu ZR, Lyu LT. Effects of Climate Change and Human Activities on Soil Erosion in the Xihe River Basin, China. Water. 2018, 10: 1085. doi: 10.3390/w10081085

[78] Wang L, Liu TT, Xie JZ. Study on the effect of different land use scenarios on runoff in Qingshuihe basin of Zhangjiakou based on SWAT model. Research of Soil and Water Conservation. 2019; 26(4): 245–251. doi: 10.13869/j.cnki.rswc.2019.04.037

[79] Li YY, Luo LF, Chang JX, Wang YM, Guo AJ, Fan JJ, et al. Hydrological drought evolution with a nonlinear joint index in regions with significant changes in underlying surface. Journal of Hydrology. 2020. 585: 124794. doi: 10.1016/j.jhydrol.2020.124794

[80] Rost S, Gerten D, Bondeau A, Lucht W, Rohwer J, Schaphoff S. Agricultural green and blue water consumption and its influence on the global water system. Water Resources Research. 2008; 44(9): W09405. doi: 10.1029/2007WR006331

[81] Du LY, Rajib A, Merwade V. Large scale spatially explicit modeling of blue and green water dynamics in a temperate mid-latitude basin. Journal of Hydrology. 2018; 562: 84–102. doi: 10.1016/j.jhydrol.2018.02.071

[82] Zhou F, Xu YP, Chen Y, Xu CY, Gao YQ, Du JK. Hydrological response to urbanization at different spatio-temporal scales simulated by coupling of CLUE-S and the SWAT model in the Yangtze River Delta region. Journal of Hydrology. 2013; 485: 113–125. doi: 10.1016/j.jhydrol.2012.12.040

[83] Ayivi F, Jha MK. Estimation of Water Balance and Water Yield in the Reedy Fork-Buffalo Creek Watershed in North Carolina using SWAT. International Soil and Water Conservation Research. 2018; 6(3): 203–213. doi: 10.1016/j.iswcr.2018.03.007

[84] Osei MA, Amekudzi LK, Wemegah DD, Preko K, Gyawu ES, Danso KO. The impact of climate and land-use changes on the hydrological processes of Owabi catchment from SWAT analysis. Journal of Hydrology: Regional Studies. 2019; 25: 100620. doi: 10.1016/j.ejrh.2019.100620

[85] Zhang H, Wang B, Liu DL, Zhang MX, Leslied LM, Yu Q. Using an improved SWAT model to simulate hydrological responses to land use change: A case study of a catchment in tropical Australia. Journal of Hydrology. 2020; 585: 124822. doi: 10.1016/j.jhydrol.2020.124822

[86] Desta H, Lemma B. SWAT based hydrological assessment and characterization of Lake Ziway sub-watersheds, Ethiopia. Journal of Hydrology: Regional Studies. 2017; 13: 122–137. doi: 10.1016/j.ejrh.2017.08.002

[87] Yang KJ, Lu CH. Evaluation of land-use change effects on runoff and soil erosion of a hilly basin-the Yanhe River in the Chinese Loess Plateau. Land Degradation and Development 2018; 29(4): 1211–1221. doi: 10.1002/ldr.2873

[88] Lian YX, Sun M, Wang JC, Luan QH, Jiao MY, Zhao XN, et al. Quantitative impacts of climate change and human activities on the runoff evolution process in the Yanhe River Basin. Physics and Chemistry of the Earth, Parts A/B/C. 2021; 122: 102998. doi: 10.1016/j.pce.2021.102998

[89] Kang YC, Gao JE, Shao H, Zhang YY, Li J, Gao Z. Evaluating the flow and sediment effects of gully land consolidation on the Loess Plateau, China. Journal of Hydrology. 2021; 600: 126535. doi: 10.1016/j.jhydrol.2021.126535

[90] Xie PX, Zhuo L, Yang X, Huang HR, Gao XR, Wu PT. Spatial-temporal variations in blue and green water resources, water footprints and water scarcities in a large river basin: A case for the Yellow River basin. Journal of Hydrology. 2020; 590: 125222. doi: 10.1016/j.jhydrol.2020.125222

[91] He YT, Li WH, Li GC, Min QW, Hai HZ. Ecological water requirement of forests in Loess Plateau. Environment Sciences. 2004, 25(3): 35–39. doi: 10.13227/j.hjkx.2004.03.007 15327249

[92] Li JY. Characteristics of vegetation ecological water demand in Yanchi County in the arid area of Central Ningxia. Arid Land Geography. 2018; 41(5): 1064–1072. doi: 10.13826/j.cnki.cn65-1103/x.2018.05.019

[93] Chu B, Shang SH. The calculation method and application of water consumption in river valley. Water resources response and sustainable utilization in a changing environment: Proceedings of academic conference on water resources committee of the China Water Conservancy Association. 2009; 12: 479–482.

[94] Yang XN, Sun WY, Li PF, Mu XM, Gao P, Zhao GJ. Integrating agricultural land, water yield and soil conservation trade-offs into spatial land use planning. Ecological Indicators. 2019; 104: 219–228. doi: 10.1016/j.ecolind.2019.04.082

[95] Luo KS, Tao FL, Moiwo JP, Xiao DP. Attribution of hydrological change in Heihe River Basin to climate and land use change in the past three decades. Nature: Scientific Reports. 2016; 6: 33704. doi: 10.1038/srep33704 27647454 PMC5028708

[96] Shao QX, Traylen A, Zhang L. Nonparametric method for estimating the effects of climatic and catchment characteristics on mean annual evapotranspiration. Water Resources Research. 2012, 48(3): W03517. doi: 10.1029/2010WR009610

[97] Wang XH, Wang BT, Xu XY, Liu T, Duan YJ, Zhao Y. Spatial and temporal variations in surface soil moisture and vegetation cover in the Loess Plateau from 2000 to 2015. Ecological Indicators. 2018; 95(1): 320–330. doi: 10.1016/j.ecolind.2018.07.058

[98] Zhu YH, Luo PP, Zhang S, Sun B. Spatiotemporal Analysis of Hydrological Variations and Their Impacts on Vegetation in Semiarid Areas from Multiple Satellite Data. Remote Sensing. 2020; 12(24): 4177. doi: 10.3390/rs12244177

[99] Liu JG, Li SX, Ouyang ZY, Tam C, Chen XD. Ecological and socioeconomic effects of China’s policies for ecosystem services. Proceeding of the National Academy of Sciences of the United States of America. 2008; 105(28): 9477–9482. doi: 10.1073/pnas.0706436105 18621700 PMC2474515

[100] Hu J, Lü YH, Fu Bj, Comber AJ, Harris P. Quantifying the effect of ecological restoration on runoff and sediment yields:A meta-analysis for the Loess Plateau of China. Progress in Physical Geography: Earth and Environment. 2017; 41(6): 753–774. doi: 10.1177/0309133317738710

